# Mutational Analysis of Rab3 Function for Controlling Active Zone Protein Composition at the *Drosophila* Neuromuscular Junction

**DOI:** 10.1371/journal.pone.0136938

**Published:** 2015-08-28

**Authors:** Shirui Chen, Hannah K. Gendelman, John P. Roche, Peter Alsharif, Ethan R. Graf

**Affiliations:** Department of Biology, Amherst College, Amherst, Massachusetts, United States of America; Columbia University, UNITED STATES

## Abstract

At synapses, the release of neurotransmitter is regulated by molecular machinery that aggregates at specialized presynaptic release sites termed active zones. The complement of active zone proteins at each site is a determinant of release efficacy and can be remodeled to alter synapse function. The small GTPase Rab3 was previously identified as playing a novel role that controls the distribution of active zone proteins to individual release sites at the *Drosophila* neuromuscular junction. Rab3 has been extensively studied for its role in the synaptic vesicle cycle; however, the mechanism by which Rab3 controls active zone development remains unknown. To explore this mechanism, we conducted a mutational analysis to determine the molecular and structural requirements of Rab3 function at *Drosophila* synapses. We find that GTP-binding is required for Rab3 to traffick to synapses and distribute active zone components across release sites. Conversely, the hydrolytic activity of Rab3 is unnecessary for this function. Through a structure-function analysis we identify specific residues within the effector-binding switch regions that are required for Rab3 function and determine that membrane attachment is essential. Our findings suggest that Rab3 controls the distribution of active zone components via a vesicle docking mechanism that is consistent with standard Rab protein function.

## Introduction

Synaptic transmission requires the regulated exocytosis of neurotransmitter-filled vesicles from axon terminals. Vesicle fusion occurs at active zones, specialized presynaptic release sites directly apposed to postsynaptic clusters of neurotransmitter receptors. Presynaptic proteins that promote vesicle release aggregate at active zones, forming the cytomatrix at the active zone (CAZ). The CAZ functions as a complex molecular machine that regulates release efficacy by controlling vesicle recruitment, the formation of the readily releasable pool, and Ca2+ channel accumulation [1]. CAZ composition and size vary across active zones, strongly correlating with site-specific release probability, and can be dynamically remodeled to alter the strength of individual synapses [2,3]. Thus, mechanisms that regulate CAZ formation and active zone protein composition are likely determinants of synapse function and may have implications for synaptic plasticity.

The *Drosophila* neuromuscular junction (NMJ) has emerged as an important model system for studying CAZ assembly. At larval NMJs, a single motor neuron forms hundreds of discrete active zones where CAZ proteins aggregate opposite postsynaptic glutamate receptor (GluR) clusters [4,5]. Constituents of the *Drosophila* CAZ are evolutionarily conserved and include Bruchpilot (Brp; the *Drosophila* homologue of ELKS/CAST/ERC) [6], RIM (Rab3 Interacting Molecule) [7], RIM-binding protein (RIM-BP) [8], Fife (the *Drosophila* homologue of Piccolo) [9], and α-liprin [10]. Ca2+ channels are also enriched at these sites via interactions with Brp, RIM, and RIM-BP [6–8,11]. Similar to vertebrate central synapses, CAZ proteins at larval NMJs are distributed heterogeneously across the entire population of active zones, resulting in sites with varying release probability [7,12,13]. What molecular mechanisms control protein composition at *Drosophila* active zones?

We previously identified the protein Rab3 as playing a novel role that dynamically controls the distribution of Brp and other CAZ components across release sites [14]. At wild type (WT) NMJs, Brp clusters at all active zones. However, in the *rab3* mutant, Brp and other constituents of the CAZ, including RIM, and Ca^2+^ channels, are enriched at one third of available sites, leaving the majority of active zones devoid of these crucial components [7,14]. The enrichment of presynaptic proteins at a fraction of sites results in larger Brp puncta that contain a greater number of Brp molecules [14,15]. While the increased size of Brp puncta in the mutant is striking, further analysis indicates that Rab3 acts to enhance the probability of initial Brp aggregation at an active zone and that increased Brp puncta size occurs secondarily [14].

Rab3 is a member of a large family of Ras-like small GTPases that are involved in vesicle trafficking and tethering to target membranes [16,17]. Rab3 itself has been extensively studied in mammals and *C*. *elegans* for its role in the synaptic vesicle cycle [18,19]. Rab3 regulates the priming of neurotransmitter-filled synaptic vesicles in a mechanism that may involve binding to its effector protein RIM [20,21]. However, RIM is not required for Rab3 to control the distribution of active zone components [7], and experiments that alter synaptic vesicle release have no effect on Brp distribution in either wild type or *rab3* mutant NMJs [14]. Thus, while Rab3 may play a role in the synaptic vesicle cycle at *Drosophila* NMJs, morphological and functional analyses suggest that Rab3 has a second separate function that initiates the clustering of Brp at nascent release sites. However, the mechanism by which Rab3 controls active zone development remains unknown. Does Rab3 operate via a molecular mechanism characteristic of general Rab function to regulate Brp distribution, or does its role in CAZ assembly implicate a novel structural mechanism atypical for Rabs?

To gain insight into how Rab3 functions to control active zone development, we conducted a mutational analysis to explore its molecular and structural requirements. We generated mutations in *Drosophila* Rab3 shown previously to disrupt distinct functional properties of its mammalian homologue, and tested whether the mutated Rab3 variants retained the ability to rescue the *rab3* synaptic phenotype. We show that GTP-binding, but not GTP hydrolysis, is necessary for Rab3 to control Brp distribution. In addition, we identify important residues in the effector-binding switch regions and determine an essential requirement for the C-terminal membrane attachment motif. Our findings are consistent with a model by which Rab3 functions to control active zone development via a vesicle associated mechanism that is typical for Rab proteins.

## Materials and Methods

### Fly Stocks

Flies were maintained at 25°C on standard fly food. The following strains were used in this study: Canton S (wildtype), *dvglut* ^NMJX^-*Gal4* [22], ELAV-*GeneSwitch* [23], *rab3* ^*rup*^ [14], PBac(PB)Rab3-GAP^c04953^ [24], Df(2R)ED2076 [14], and Df(2L)ED775 [24]. The P-element and deficiency lines were obtained from the Bloomington Stock Center. For expression of UAS-tagged transgenes driven by ELAV-*GeneSwitch*, females were maintained on fly food containing 25μg/ml RU486 (Mifepristone; Sigma, St. Louis, MO) for 2 days before mating and allowed to lay eggs on RU486-containing fly food where larvae were then maintained until dissection.

### Transgenic Constructs

The wild type *UAS-rab3* transgene was generated by subcloning the *rab3* cDNA (LP05860, *Drosophila* Genomics Resource Center, Bloomington, IN) into a pUASTattB vector [25]. The *UAS-rab3ΔC* transgene with a deletion of the last three C-terminal amino acids was created by subcloning the *rab3* cDNA into the pUASTattB vector using a reverse primer that incorporated a premature stop sequence. All other mutant *UAS-rab3* transgenes were created with the Stratagene (La Jolla, CA) Site-Directed Mutagenesis kit. Primer sequences used to create the wild type and mutant UAS-tagged transgenes are as follows: *UAS-rab3* (forward: 5’-GGTAGAATTCATGGCGAGTGGCGGAGACCCC-3’ and reverse: 5’-GTCAGCGGCCGCCTAACAATTGCAGTTGGCATTAGGC-3’), *UAS-rab3ΔC* (forward: 5’-GGTAGAATTCATGGCGAGTGGCGGAGACCCC-3’ and reverse: 5’-GTTAGCGGCCGCCTAGTTGGCATTAGGCGTGCCCTG-3’), *UAS-rab3T35N* (forward: 5’-GGCAACTCCAGCGTGGGCAAGAACAGCTTCCTCTTCCGC-3’ and reverse: 5’-GCGGAAGAGGAAGCTGTTCTTGCCCACGCTGGAGTTGCC-3’), *UAS-rab3N134I* (forward: 5’-GTGATCCTGGTGGGCATCAAGTGCGACATGGAG-3’ and reverse: 5’-CTCCATGTCGCACTTGATGCCCACCAGGATCAC-3’), *UAS-rab3Q80L* (forward: 5’-GACACTGCTGGACTGGAGCGGTACAGAACTATC-3’ and reverse: 5’-GATAGTTCTGTACCGCTCCAGTCCAGCAGTGTC-3’), *UAS-rab3F50A* (forward: 5’-GCTTCACATCCGCCGCGGTCTCCACGGTGGG-3’ and reverse: 5’-CCCACCGTGGAGACCGCGGCGGATGTGAAGC-3’), *UAS-rab3V51A* (forward: 5’-CTTCACATCCGCCTTCGCGTCCACGGTGGGCATTG-3’ and reverse: 5’-CAATGCCCACCGTGGACGCGAAGGCGGATGTGAAG-3’), *UAS-rab3S52A* (forward: 5’-CATCCGCCTTCGTCGCGACGGTGGGCATTG-3’ and reverse: 5’-CAATGCCCACCGTCGCGACGAAGGCGGATG-3’), *UAS-rab3T53A* (forward: 5’-GCCTTCGTCTCCGCGGTGGGCATTGAC-3’ and reverse: 5’-GTCAATGCCCACCGCGGAGACGAAGGC-3’), *UAS-rab3F58S* (forward: 5’-GGTGGGCATTGACTCTAAGGTGAAGACCG-3’ and reverse: 5’-CGGTCTTCACCTTAGAGTCAATGCCCACC-3’), *UAS-rab3R82A* (forward: 5’-CTGCTGGACAGGAGGCGTACAGAACTATCAC-3’ and reverse: 5’-GTGATAGTTCTGTACGCCTCCTGTCCAGCAG-3’), *UAS-rab3Y83A* (forward: 5’-GGACAGGAGCGGGCGAGAACTATCACC-3’ and reverse: 5’-GGTGATAGTTCTCGCCCGCTCCTGTCC-3’), *UAS-rab3FDY18-20AAA* (forward: 5’-AGAAGGATGCCGCCGACCAGAACGCTGCCGCCATGTTCAAGCTGCTCATCATTG-3’ and reverse: 5’-CAATGATGAGCAGCTTGAACATGGCGGCAGCGTTCTGGTCGGCGGCATCCTTCT-3’), *UAS-rab3WDN124-126AAA* (forward: 5’-GGTGACACAGATCAAAACGTATTCGGCGGCCGCTGCCCAGGTGATCCTGG-3’ and reverse: 5’-CCAGGATCACCTGGGCAGCGGCCGCCGAATACGTTTTGATCTGTGTCACC-3’), *UAS-rab3KM185-185AA* (forward: 5’-GTGGACATCATCTGCGATGCGGCGTCCGAGAGCCTGGACG-3’ and reverse: 5’-CGTCCAGGCTCTCGGACGCCGCATCGCAGATGATGTCCAC-3’), *UAS-rab3SL189-190AA* (forward: 5’-GCGATAAGATGTCCGAGGCGGCGGACGCGGATCCGACGTTAG-3’ and reverse: 5’-CTAACGTCGGATCCGCGTCCGCCGCCTCGGACATCTTATCGC-3’). Mutations were confirmed by sequencing. All transgenes were inserted into the attP landing site of the M{vas-int.Dm]ZH-2A; M[3xP3-RFP.attp]ZH-86Fb fly strain using the phi-C31 system [25]. Transgenic flies were generated by BestGene, Inc.

### Immunohistochemistry

Third-instar larvae were dissected in PBS and fixed in either Bouin’s fixative for 5 min. Larvae were washed with PBS containing 0.1% Triton X-100 (PBT) and blocked in 5% NGS in PBT for 30 min, followed by overnight incubation in primary antibodies in 5% NGS in PBT, three washes in PBT, incubation in secondary antibodies in 5% NGS in PBT for 45 min, three final washes in PBT, and equilibration in 70% glycerol in PBS. Samples were mounted in VectaShield (Vector, Burlingame, CA). The following primary antibodies were used: mouse α-Brp, 1:250 (Developmental Studies Hybridoma Bank), rabbit α-DGluRIII, 1:2500 [26], and rabbit α-Rab3, 1:1000 [14]. Goat Cy5- and Cy3- conjugated secondary antibodies against mouse and rabbit IgG and Cy5-conjugated goat α-HRP were used at 1:1000 and were obtained from Jackson ImmunoResearch. Antibodies obtained from the Developmental Studies Hybridoma Bank were developed under the auspices of the National Institute of Child Health and Human Development and maintained by the Department of Biological Sciences of the University of Iowa, Iowa City, IA.

### Imaging and analysis

Samples were imaged using a Nikon (Tokyo, Japan) C2 confocal microscope. All genotypes for an individual experiment were imaged at the same gain and set such that signals from the brightest genotype for a given experiment were not saturating. Only NM4b NMJs on muscle 4 were analyzed. NMJs in segments 2 through 6 were imaged for rescue experiments utilizing the *dvglut* ^NMJX^-*Gal4* driver. NMJs in segments 5 and 6 were imaged for dominant negative experiments utilizing the ELAV-*GeneSwitch* driver. Images were analyzed using MetaMorph software (Molecular Devices, Sunnyvale, CA). Statistical analysis was performed using ANOVA for comparison of samples within an experimental group. All histograms and measurements are shown as mean±SEM.

To determine the percentage of GluR clusters apposed by Brp, Brp and DGluRIII puncta were manually counted, and DGluRIII clusters that were not opposite to a detectable Brp punctum were counted as unapposed DGluRIII clusters. MetaMorph software was used for the quantification of Brp puncta size and Rab3 average intensity. For measurement of Brp area, thresholds were kept constant across all genotypes for a given experiment. Although most Brp puncta were distinct, occasional overlapping puncta were separated with the cut drawing tool. For measurements of Rab3 intensity, the area of the NMJ was first defined by HRP and Brp signal. The average intensity of Rab3 signal within each defined NMJ was then calculated, and the average muscle background intensity was subtracted.

### Electrophysiology

Two Electrode Voltage clamp recordings were done in muscle 6 of abdominal segments A3 and A4 of wandering third instar larvae. Electrodes with resistances between 10 and 20 MΩ were used. The cells were held at −70 mV in a modified HL-3 saline solution [27] containing the following (in mM): 70 NaCl, 5 KCl, 10 NaHCO_3_, 115 Sucrose, 5 Trehalose, 5 HEPES, 0.4 CaCl, and 10 MgCl. Recordings were done in voltage clamp mode using an AxoClamp 2B Amplifier (Axon Instruments) low pass filtered at 1kHz, and digitized at 10kHz with an Instrutech ITC-18 computer interface using Patchmaster Software (HEKA Electronics). Cells requiring 1 nA or more holding current were discarded. Spontaneous events were recorded for 1 min and the average amplitude and frequency of the spontaneous events was quantified using Mini Analysis Software (Synaptosoft Inc.). For stimulated EJCs, AgCl2 wire in a glass suction electrode was used to stimulate the cut end of the segmental nerve for 1 ms at 1.5x the threshold voltage. A Master 8 stimulator and Isoflex stimulation isolation unit (A.M.P.I.) were used to control the duration and amplitude of the stimulation. The elicited currents from 10 successive stimulation protocols were averaged offline using custom programs written by Josef Trapani (Amherst College) in Igor Pro software (WaveMetrics Inc.). For stimulus trains, the nerve was stimulated with 10 trains of 5 pulses at 20 Hz with 5 seconds rest intervals between trains. The 10 trains were averaged and the amplitudes of the 1st and 5th pulse were used to calculate the facilitation index.

## Results

### Generation of Mutant Rab3 Transgenes

**Fig 1:**
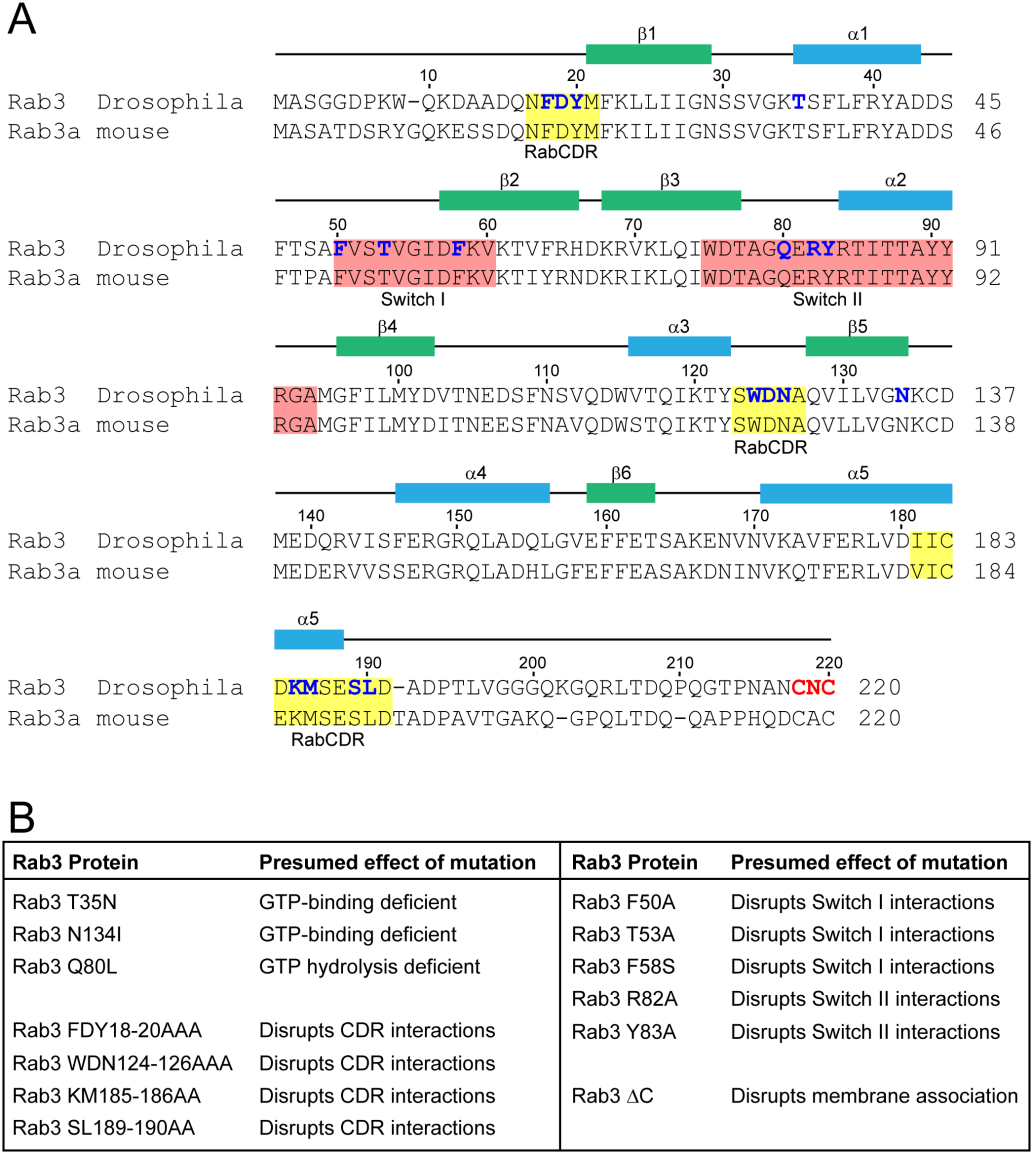
Rab3 sequence and list of mutations analyzed. (A) Sequence alignment of *Drosophila* Rab3 with mouse Rab3a. Amino acid numbers and labels for secondary structures (green, β-strands; blue, α-helices) and switch regions (pink) are shown for *Drosophila* Rab3, as determined by crystal structure analysis of fly Rab3 [29]. CDR region labels (yellow) correspond to the RabCDRs identified in rodent Rab3 [34]. Amino acids labelled in blue correspond to the residues studied in the mutational analysis. Amino acids labelled in red correspond to the three residues deleted following C-terminal truncation. (B) List of the mutant variants of *Drosophila* Rab3 generated for this study and the presumptive effect of each mutation.

To investigate the molecular mechanism by which Rab3 functions to control the protein composition of individual active zones we performed a mutational analysis of *Drosophila* Rab3. Rab3 is highly conserved between flies and rodents both in terms of amino acid sequence (78% sequence identity with mouse Rab3a [28]; Fig 1A) and protein structure [29]. Previous studies have identified specific residues of rodent Rab3 required for its roles in the regulation of exocytosis and the trafficking of synaptic vesicles [28,30–34]. Using these mammalian structure-function and biochemical studies as a guide, we designed homologous mutations in *Drosophila* Rab3 that disrupt various functional properties of the protein, including GTP/GDP cycling, protein-protein interactions, and membrane association (Fig 1). To determine how the mutations affect Rab3 function at the Drosophila NMJ, we utilized the UAS/Gal4 system to drive expression of each variant in larval neurons. Transgenic flies containing *UAS*-*rab3* transgenes were generated by site-specific integration utilizing the phi-C31 system to avoid variability of transgene expression due to positional effects [25]. *UAS-rab3* transgenes were expressed in the *rab3* ^*rup*^ */* Df(2R)ED2076 mutant background via the neuronal *dvglut* ^NMJX^-*Gal4* driver. The *rab3* ^*rup*^ allele contains a 5 base pair deletion near the 3’ end of the gene and behaves as a genetic null [14]. Mutant variants of Rab3 were assayed for their ability to rescue the *rab3* synaptic phenotype when expressed in the mutant background, as described below.

### GTP-Binding is Required for Rab3 to Control Active Zone Composition

Rab3 is a small GTPase that switches between an activated GTP-bound and de-activated GDP-bound state, which determines the binding partners and function of the protein [17,19]. Cycling between these states is regulated by Rab3-GAP (GTPase Accelerating Protein) that enhances the hydrolysis of GTP to GDP and Rab3-GEF (Guanine nucleotide Exchange Factor) that stimulates release of GDP to allow for the binding of a new GTP molecule [35]. Rab3 must be GTP-bound to associate with effector proteins such as RIM [21], making GTP-binding essential for its roles in exocytosis and synaptic vesicle trafficking. Is GTP-binding required in a similar manner for Rab3 to control the protein composition of active zones? To analyze the requirement of GTP-binding, we tested the function of Rab3T35N and Rab3N134I. Studies of homologous mutations in rodent Rab3 indicate that Rab3T35N only binds GDP [31] while Rab3N134I has a high dissociation rate for both GTP and GDP [30]. We previously showed that neuronal expression of Rab3N134I fails to rescue the *rab3* mutant phenotype [14]. However, since the two mutations have differing effects on nucleotide binding, and Rab3N134I may still be predominantly GTP-bound even though it is defective in binding both GTP and GDP [30], we wished to test the function of GTP-binding defective Rab3 in more detail.

**Fig 2:**
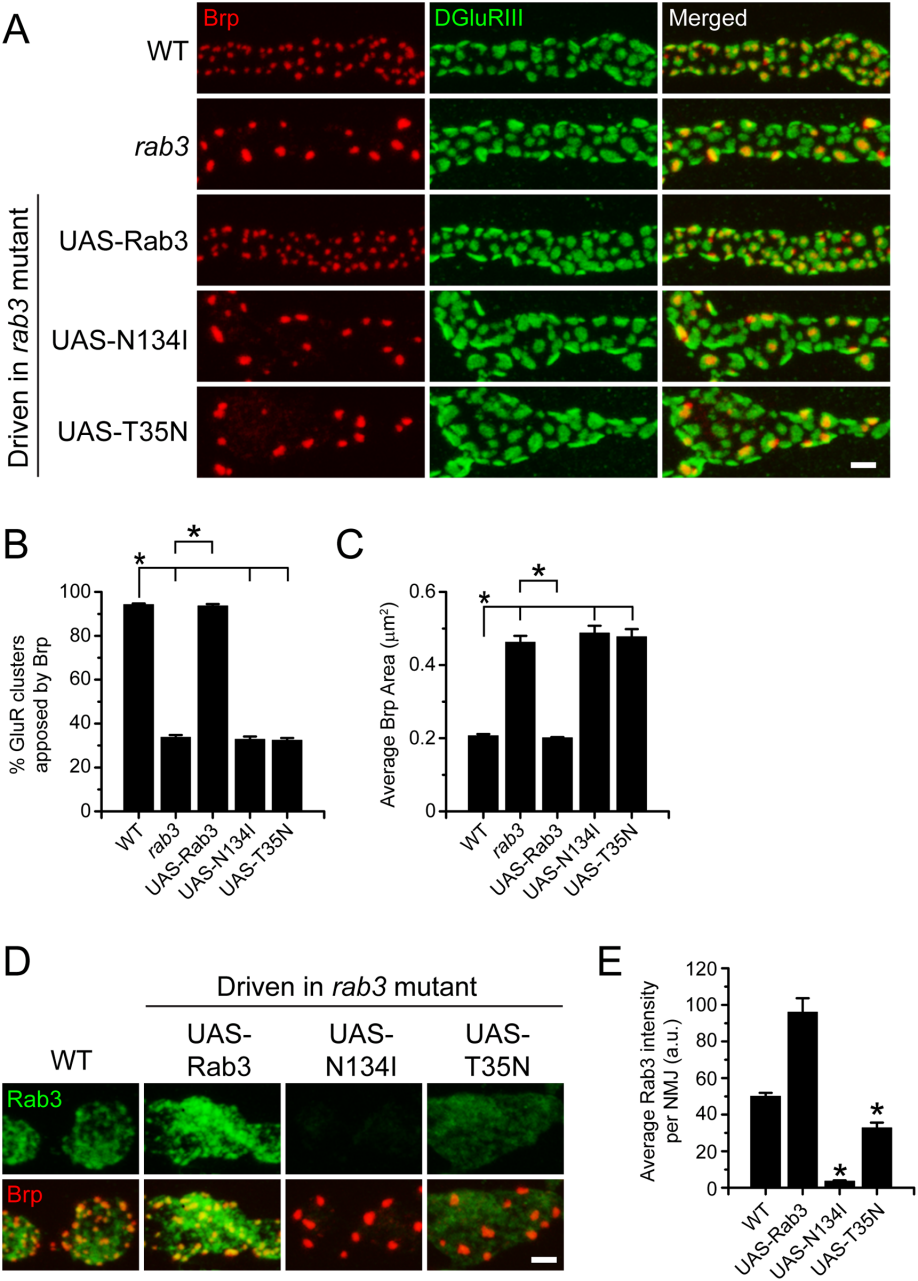
GTP-binding is required for Rab3 function and transport to NMJs. (A) Images of muscle 4 NMJs stained for the presynaptic active zone protein Brp (red) and the postsynaptic receptor DGluRIII (green) from WT (*dvglut* ^NMJX^-*Gal4*/+), the *rab3* mutant (*dvglut* ^NMJX^-*Gal4*/+; *rab3* ^*rup*^/Df(2R)ED2076), the *rab3* mutant expressing the wild type *UAS-rab3* transgene (*dvglut* ^NMJX^-*Gal4*/+; *rab3* ^*rup*^/Df(2R)ED2076; *UAS-rab3*/+), the *rab3* mutant expressing the *UAS-rab3N134I* transgene (*dvglut* ^NMJX^-*Gal4*/+; *rab3* ^*rup*^/Df(2R)ED2076; *UAS-rab3N134I*/+), and the *rab3* mutant expressing the *UAS-rab3T35N* transgene (*dvglut* ^NMJX^-*Gal4*/+; *rab3* ^*rup*^/Df(2R)ED2076; *UAS-rab3T35N*/+). Scale bar, μm. (B-C) Histograms show (B) the average percentage of DGluRIII clusters apposed to Brp puncta per NMJ and (C) the average area of individual Brp puncta for the genotypes listed in (A). n = 10 NMJs for all genotypes; *p<<0.001. (D) Images of NMJs costained with α-Rab3 (green) and α-Brp (red) from WT (*dvglut* ^NMJX^-*Gal4*/+), the *rab3* mutant expressing the wild type *UAS-rab3* transgene (*dvglut* ^NMJX^-*Gal4*/+; *rab3* ^*rup*^/Df(2R)ED2076; *UAS-rab3*/+), the *rab3* mutant expressing the *UAS-rab3N134I* transgene (*dvglut* ^NMJX^-*Gal4*/+; *rab3* ^*rup*^/Df(2R)ED2076; *UAS-rab3N134I*/+), and the *rab3* mutant expressing the *UAS-rab3T35N* transgene (*dvglut* ^NMJX^-*Gal4*/+; *rab3* ^*rup*^/Df(2R)ED2076; *UAS-rab3T35N*/+). Scale bar, 2μm. (E) Histogram shows average intensity of Rab3 per NMJ for the genotypes listed in (D). n = 10 NMJs for all genotypes; *p<<0.001 versus WT and expression of the wild type *UAS-rab3* transgene.

We used *dvglut* ^NMJX^-*Gal4* to drive expression of *UAS-rab3N134I* and *UAS-rab3T35N* in the *rab3* mutant and assayed for Brp distribution across active zones. At larval NMJs, the essential glutamate receptor subunit DGluRIII is a good marker for the location of presynaptic active zones as it localizes in clusters opposite release sites [26]. At wild type NMJs, Brp is localized to almost all active zones such that nearly all postsynaptic GluR clusters are apposed to Brp. However, in the *rab3* mutant only about one third of GluR clusters are apposed to Brp and average Brp puncta size is twice as large as compared to wild type (Fig 2A–2C). In agreement with previous studies, neuronal expression of the wild type *UAS-rab3* transgene in the *rab3* mutant fully rescues Brp distribution. Conversely, expression of either *UAS-rab3N134I* or *UAS-rab3T35N* in the *rab3* mutant fails to rescue the mutant phenotype (Fig 2A–2C). The percentage of GluR clusters apposed to Brp is indistinguishable between *rab3* mutant larvae that express *UAS-rab3N134I* and *UAS-rab3T35N* and the *rab3* mutant itself. Likewise, average Brp puncta size is identical between *rab3* mutants that express no *UAS* transgene and *rab3* mutants that express *rab3N134I* and *UAS-rab3T35N*.

The fact that neither GTP-binding defective version of Rab3 can rescue the mutant phenotype suggests that GTP-binding is required for Rab3 function at the NMJ. However, studies in cultured mouse neurons indicate that Rab3 must be GTP-bound for proper axonal trafficking of Rab3 to the synapse [36]. Could the lack of rescue be due to defective transport of Rab3N134I and Rab3T35N to the NMJ? To determine whether the GTP-binding defective variants of Rab3 localize to NMJs, we immunostained NMJs with an antibody against *Drosophila* Rab3 that recognizes both endogenous and transgenic Rab3 but for which staining is absent in the *rab3* mutant [14]. This antibody recognizes an epitope that is present and unmodified in all transgenic Rab3 proteins analyzed in this study. As described previously [14], in wild type NMJs, Rab3 localizes throughout the NMJ in a relatively diffuse pattern reminiscent of synaptic vesicle proteins but with concentrations of protein at active zones that are visualized as Rab3 puncta that colocalize with Brp. A similar Rab3 localization pattern is observed at wild type NMJs and *rab3* mutant NMJs that transgenically express wild type *UAS-Rab3* (Fig 2D), although, transgenic expression of *UAS-Rab3* when driven by *dvglut* ^NMJX^-*Gal4* in *rab3* mutants results in Rab3 protein levels at the NMJ that are significantly higher than endogenous Rab3 (p<<0.001). Conversely, when the GTP-binding defective variants are driven in *rab3* mutant neurons we observe almost no Rab3N134I protein and defective but visible accumulation of Rab3T35N protein (Fig 2D). In addition, Rab3T35N that does accumulate at the NMJ fails to aggregate at active zones. Quantification of average intensity of Rab3 protein per NMJ indicates a significant reduction of Rab3N134I and Rab3T35N as compared to both transgenic Rab3 and endogenous Rab3 (Fig 2E). While Rab3N134I and Rab3T35N protein levels at the NMJ are reduced as compared to endogenous Rab3, Rab3T35N is still present with average staining intensity that is 65% of endogenous Rab3. The synaptic phenotype is not observed in *rab3* heterozygotes, so a reduction that is less than 50% should have little effect. It is unlikely that Rab3N134I could rescue synaptic development due to its absence at the NMJ. However, the presence of Rab3T35N at the NMJ, albeit reduced, combined with the complete lack of rescue, suggests that GTP-binding is required for Rab3 to control active zone development.

**Fig 3:**
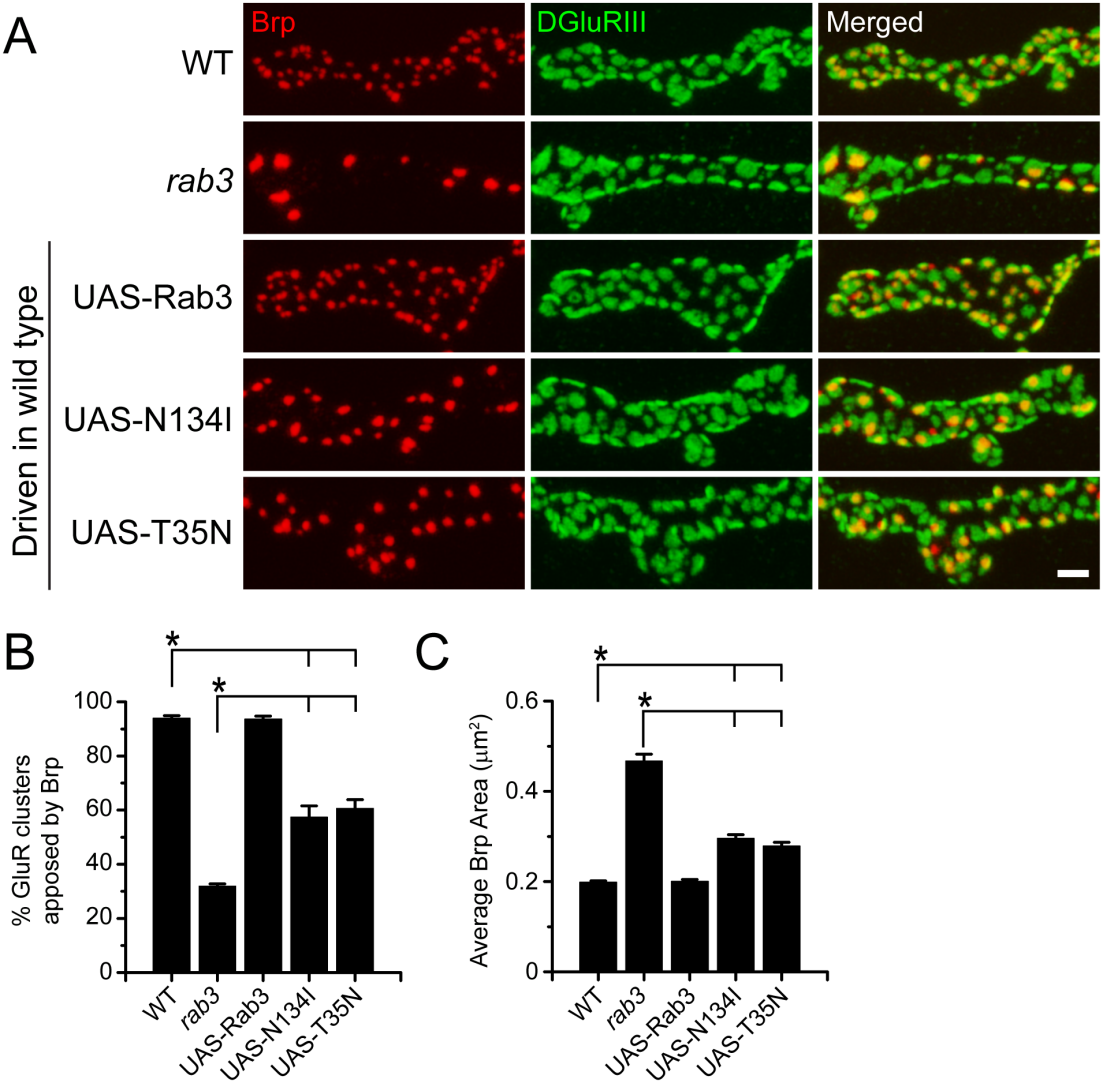
GTP-binding defective variants of Rab3 act as dominant negatives. (A) Images of NMJs costained with α-Brp (red) and α-DGluRIII (green) from WT (ELAV-*GeneSwitch*/+), the *rab3* mutant (*rab3* ^*rup*^/Df(2R)ED2076; ELAV-*GeneSwitch*/+), the wild type *UAS-rab3* transgene expressed in a wild type background (ELAV-*GeneSwitch*/*UAS-rab3*), the *UAS-rab3N134I* transgene expressed in a wild type background (ELAV-*GeneSwitch*/*UAS-rab3N134I*), and the *UAS-rab3T35N* transgene expressed in a wild type background (ELAV-*GeneSwitch*/*UAS-rab3N134I*). Scale bar, 2μm. (B-C) Histograms show (B) the average percentage of DGluRIII clusters apposed to Brp puncta per NMJ and (C) the average area of individual Brp puncta for the genotypes listed in (A). n = 10 NMJs for all genotypes; *p<<0.001.

Since the defective accumulation of Rab3N134I and Rab3T35N compromises their utilization in a rescue assay, we further explored the requirement of GTP-binding for Rab3 function in synapse development by testing whether Rab3N134I and Rab3T35N can affect active zone composition in a dominant negative fashion. In other systems, GTP-binding defective variants of Rab3 act as dominant negatives [37,38], likely by binding to and inhibiting proteins involved in the GDP/GTP cycle such as Rab3-GEF [31,37]. To achieve a dominant negative effect, we used *ELAVGeneSwitch-Gal4* to drive the *UAS-Rab3* transgenes at high levels in a wild type background. Both Rab3N134I and Rab3T35N produce a hypomorphic *rab3* phenotype when expressed in wild type (Fig 3). Approximately 60% of GluR clusters are apposed to Brp following Rab3N134I and Rab3T35N expression, a phenotype that is less severe than the *rab3* mutant where only one third of GluR clusters are Brp-positive but a result that is significantly different than wild type (Fig 3B). In addition, average Brp puncta area is greater than wild type following Rab3N134I and Rab3T35N expression, although the increase in Brp puncta size is not as extreme as is observed in the *rab3* mutant (Fig 3C). Importantly, this effect is due to the expression of GTP-binding defective variants of Rab3 and not the overexpression of Rab3 itself as overexpression of the wild type *UAS-rab3* transgene has no effect. Interestingly, this dominant negative effect was only observed in NMJs associated with more posterior segments of the larvae, perhaps due to variations in expression strength or developmental timing associated with driving transgene expression via *ELAVGeneSwitch-Gal4*. Alternatively, Rab3N134I and Rab3T35N expression may cause axon transport defects of endogenous Rab3 that has greater impact in the longer motor neurons associated with posterior segments. As a result only NMJs in segments 5 and 6 were analyzed. Taken together, the rescue and dominant negative experiments indicate that Rab3 must be able to bind GTP to control active zone composition.

### GTP Hydrolysis is Unnecessary for Rab3 Rescue

**Fig 4:**
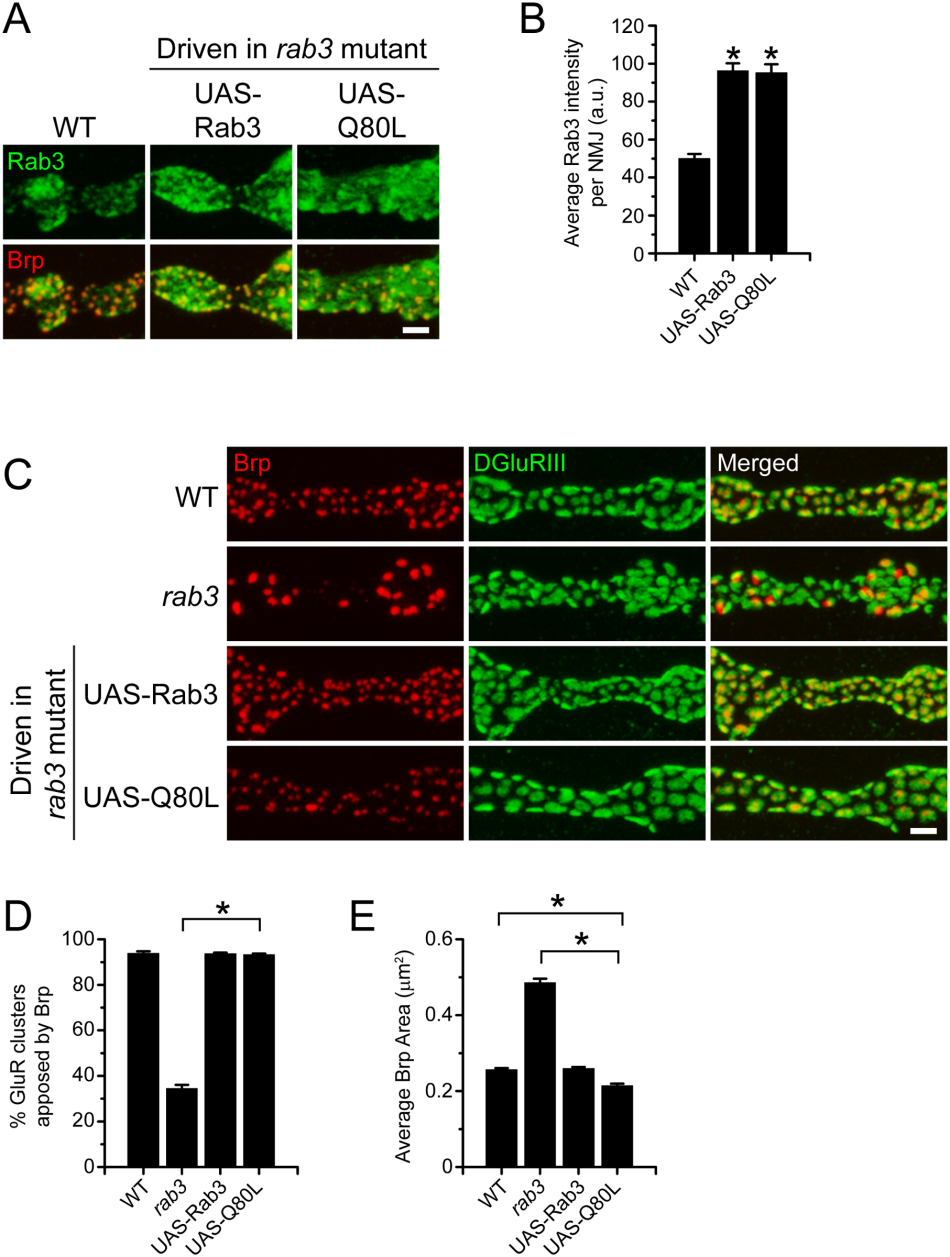
GTP hydrolysis defective Rab3 rescues Brp distribution in the *rab3* mutant. (A) Images of NMJs costained with α-Rab3 (green) and α-Brp (red) from WT (*dvglut* ^NMJX^-*Gal4*/+), the *rab3* mutant expressing the wild type *UAS-rab3* transgene (*dvglut* ^NMJX^-*Gal4*/+; *rab3* ^*rup*^/Df(2R)ED2076; *UAS-rab3*/+), and the *rab3* mutant expressing the *UAS-rab3Q80L* transgene (*dvglut* ^NMJX^-*Gal4*/+; *rab3* ^*rup*^/Df(2R)ED2076; *UAS-rab3Q80L*/+). Scale bar, 2μm. (B) Histogram shows average intensity of Rab3 per NMJ for the genotypes listed in (A). n = 10 NMJs for all genotypes; *p<<0.001 versus WT. (C) Images of NMJs costained with α-Brp (red) and α-DGluRIII (green) from WT (*dvglut* ^NMJX^-*Gal4*/+), the *rab3* mutant (*dvglut* ^NMJX^-*Gal4*/+; *rab3* ^*rup*^/Df(2R)ED2076), the *rab3* mutant expressing the wild type *UAS-rab3* transgene (*dvglut* ^NMJX^-*Gal4*/+; *rab3* ^*rup*^/Df(2R)ED2076; *UAS-rab3*/+), and the *rab3* mutant expressing the *UAS-rab3Q80L* transgene (*dvglut* ^NMJX^-*Gal4*/+; *rab3* ^*rup*^/Df(2R)ED2076; *UAS-rab3Q80L*/+). Scale bar, 2μm. (D-E) Histograms show (D) the average percentage of DGluRIII clusters apposed to Brp puncta per NMJ and (E) the average area of individual Brp puncta for the genotypes listed in (C). n = 10 NMJs for all genotypes; *p<<0.001.

As a GTPase, Rab3 can hydrolyze its bound GTP to GDP and inactivate itself [35], a function that is enhanced by Rab3-GAP [35]. Is the GTP hydrolysis activity of Rab3 required for it to control active zone development? To analyze the necessity of GTP hydrolysis, we studied the function of Rab3Q80L which incorporates a mutation that inhibits the GTPase activity of Rab3 even in the presence of the GAP [30] and acts as a constitutively active variant of Rab3. To examine the function of Rab3Q80L, we used *dvglut* ^NMJX^-*Gal4* to drive expression of *UAS-rab3Q80L* in the *rab3* mutant. Unlike the GTP-binding defective variants of Rab3, Rab3Q80L was trafficked appropriately to the NMJ, resulting in an average intensity of α-Rab3 signal that was higher than endogenous Rab3 but equivalent to wild type Rab3 driven by the *UAS-rab3* transgene (Fig 4A and 4B). In addition, the staining pattern of Rab3Q80L at the NMJ was similar to that observed for wild type Rab3 with aggregations of Rab3Q80L colocalized with Brp puncta. Furthermore, analysis of Brp distribution at *rab3* mutant NMJs expressing Rab3Q80L by costaining with α-Brp and α-DGluRIII revealed that Rab3Q80L is able to rescue the *rab3* mutant phenotype (Fig 4C). The percentage of GluR clusters apposed to Brp following Rab3Q80L expression is significantly greater than in the *rab3* mutant and identical to wild type (Fig 4D). Interestingly, while Rab3Q80L expression also rescues Brp size, average Brp area is modestly smaller than wild type (Fig 4E). These results suggest that Rab3Q80L may have a gain-of-function effect that causes Brp puncta to be even smaller than normal. To explore this gain-of-function effect further, we tested whether expression of Rab3Q80L in a wild type background with the the *ELAVGeneswitch* driver also caused reduced Brp size. Whereas expression of unmutated *UAS-Rab3* had no effect on Brp size, expression of *UAS-Rab3Q80L* again resulted in a mild but significant reduction in average Brp size compared to wild type (WT, 0.21 ± 0.01 μm^2^, n = 10; *UAS-Rab3* driven in WT, 0.21 ± 0.01 μm^2^, n = 10; *UAS-Rab3Q80L* driven in WT, 0.17 ± 0.01 μm^2^, n = 10; p<<0.001 for *UAS-Rab3Q80L* vs. both WT and UAS-Rab3; analysis restricted to segments 5 and 6 as described above).

Since hydrolysis-defective Rab3 decreases Brp puncta size, we asked whether a similar Brp phenotype is observed in a *rab3-GAP* mutant. Rab3-GAP regulates Rab3 by enhancing the hydrolytic function of Rab3 and converting it to its GDP-bound state [39]. Thus, defects in Rab3-GAP result in the accumulation of the GTP-bound form [40], similar to the expression of Rab3Q80L. Previous studies of a *Drosophila rab3-GAP* mutant show normal Brp number and density at *rab3-GAP* mutant NMJs [24], consistent with NMJs that express Rab3Q80L.To determine whether the gain-of-function phenotype observed following Rab3Q80L expression is also present in *rab3-GAP* mutant NMJs, we further analyzed Brp size. Measurements of average Brp area indicate that Brp size in *rab3-GAP* mutants is the same as wild type (CS, 0.22 ± 0.01 μm^2^, n = 10; *rab3-GAP* ^*c04953*^/Df(2L)ED775, 0.22 ± 0.01 μm^2^, n = 10; p>0.9), indicating that *rab3-GAP* mutation does not phenocopy Rab3Q80L overexpression.

**Fig 5:**
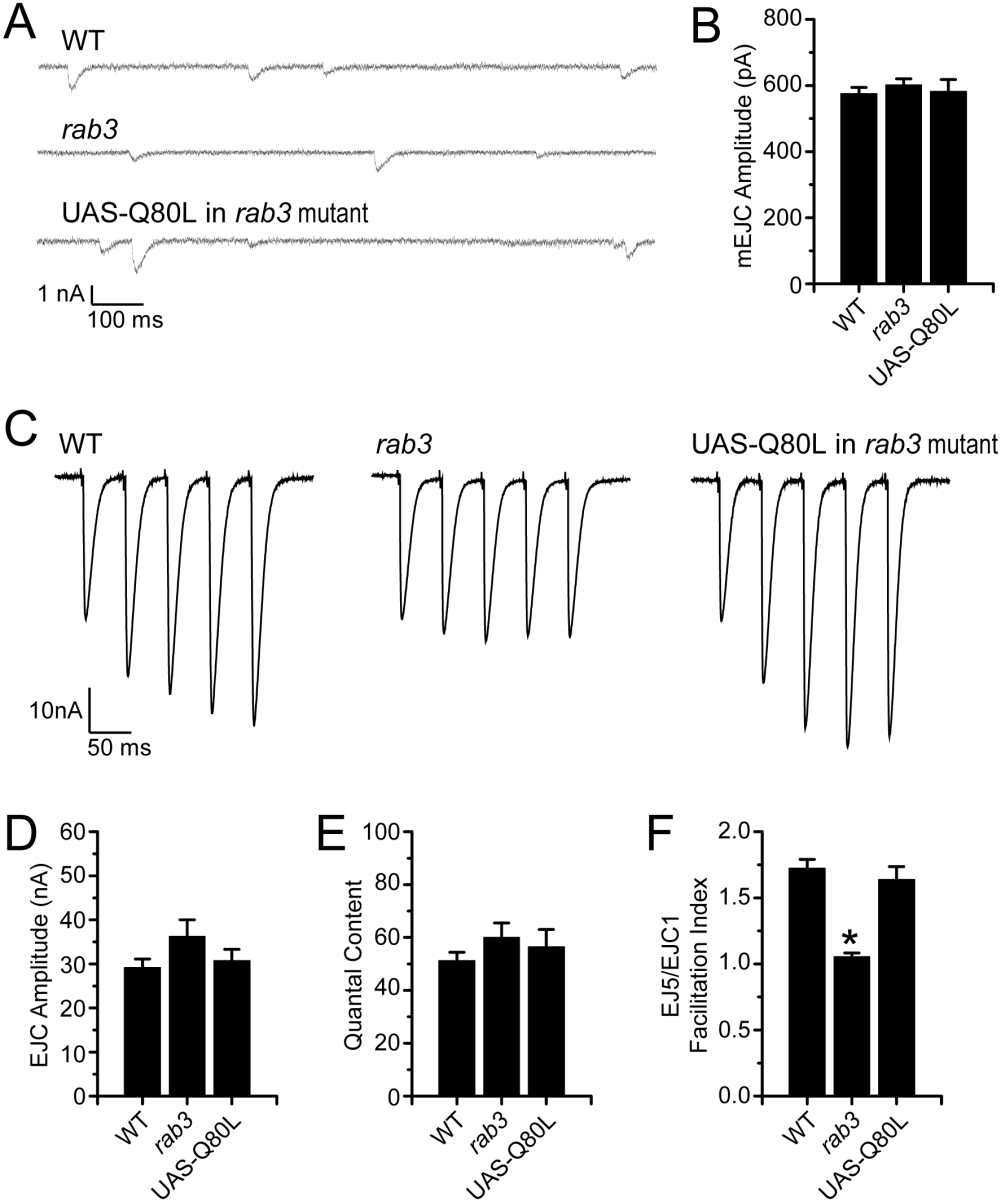
GTP hydrolysis defective Rab3 rescues the short-term facilitation deficits of the *rab3* mutant. (A) Representative mEJC traces from WT (*dvglut* ^NMJX^-*Gal4*/+), the *rab3* mutant (*dvglut* ^NMJX^-*Gal4*/+; *rab3* ^*rup*^ */* Df(2R)ED2076), and the *rab3* mutant expressing the *UAS-rab3Q80L* transgene (*dvglut* ^NMJX^-*Gal4*/+; *rab3* ^*rup*^/Df(2R)ED2076; *UAS-rab3Q80L*/+). (B) Histogram shows average mEJC amplitude for the genotypes listed in (A). WT, n = 24; *rab3* mutant, n = 23; *rab3* mutant with Rab3Q80L, n = 16. (C) Representative EJC traces of a train of five stimuli given at a frequency of 20 Hz in 0.40 mM Ca^2+^ from the genotypes listed in (A), stimulation artifact removed for clarity. (D-E) Histograms showing (D) average evoked EJC amplitude from a single stimulus and (E) estimates of quantal content, calculated by dividing average evoked EJC amplitude by average mEJC amplitude, for each of the three genotypes. WT, n = 22; *rab3* mutant, n = 22; *rab3* mutant with Rab3Q80L, n = 16. (F) Histogram showing quantification of the average facilitation index (FI) for each of the three genotypes, calculated by dividing the amplitude of the fifth EJC by the amplitude of the first EJC in a 20 Hz stimulus train. WT, n = 20; *rab3* mutant, n = 22; *rab3* mutant with Rab3Q80L, n = 13; *p<<0.001 versus WT and rescue with *UAS-rab3Q80L*.

Even though we observe a mild gain-of-function phenotype concerning average Brp size, Rab3Q80L rescues Brp distribution in the *rab3* mutant. Can Rab3Q80L also rescue the functional deficits observed in the mutant? We have previously shown that *rab3* disruption has no effect on the spontaneous release of vesicles and that evoked vesicle release is normal in the mutant following a single action potential [14]. However, *rab3* mutation leads to defective short-term facilitation which is rescued by expression of a wild type *UAS-rab3* transgene in the mutant [14]. To determine the effect of Rab3Q80L on NMJ function, we performed voltage-clamp recordings of muscle 6 NMJs from segments A3 and A4 in wild type larvae, *rab3* mutants, and *rab3* mutants expressing Rab3Q80L. Spontaneous miniature excitatory junctional current (mEJC) amplitude recorded from *rab3* mutant NMJs expressing Rab3Q80L is identical to both wild type and the *rab3* mutant mEJC amplitude (Fig 5A and 5B). In addition, excitatory junctional current (EJC) amplitude evoked by a single action potential in 0.4 mM Ca^2+^ is similar between the three genotypes (Fig 5C, first pulse, and Fig 5D). Thus, the number of vesicles released following an action potential as estimated by quantal content (EJC/mEJC) is comparable between wild type, *rab3* mutant NMJs, and the *rab3* mutant NMJs expressing Rab3Q80L (Fig 5E), indicating that Rab3Q80L expression does not cause gross changes in vesicle release.

We next tested whether Rab3Q80L is able to rescue the defective short-term facilitation that has been observed in the *rab3* mutant. In low extracellular calcium, trains of pulses with a short interpulse interval typically result in facilitation at wild type NMJs whereby subsequent EJCs increase in size. This is often due to a build-up of residual calcium and is indicative of NMJs that have sites with a low probability of release [41]. However, the altered distribution of active zone components in the *rab3* mutant gives rise to a small number of functional sites that have high release probability [13,14]. This results in reduced facilitation that is indicative of high probability sites. To determine if Rab3Q80L is able to rescue the defective short-term facilitation observed in the mutant we induced release with a train of stimuli evoked at a frequency of 20 Hz in 0.4 mM Ca^2+^ and calculated the facilitation index (FI) by dividing the amplitude of the fifth EJC by that of the first EJC (Fig 5C and 5F). Consistent with previous findings, whereas subsequent evoked events are larger following repeated stimulation in wild type, facilitation is impaired in the *rab3* mutant. Conversely, when Rab3Q80L is expressed in the *rab3* mutant, facilitation is rescued and the FI is restored to wild type levels (Fig 5F). Thus, Rab3Q80L can rescue both the morphological and electrophysiological defects of the *rab3* mutant, indicating that GTP hydrolysis is not required for Rab3 to control NMJ development and function.

### The Switch Regions are Required for Rab3 Function

We show above that GTP-binding is required for Rab3 function at the NMJ. Since the GTP- versus GDP- bound state of Rab3 determines its protein binding properties [21,35,42], GTP-binding is likely required for Rab3 to interact with effector proteins to control the protein composition of active zones. However, the proteins that Rab3 must bind to control active zone development remain unclear. We have previously shown that the Rab3 effector protein RIM is not required for Rab3 to control Brp distribution at the NMJ [7]. Another potential effector is Rabphilin, a Rab3-binding protein that regulates neurotransmitter release by contributing to SNARE function [43]. However, no mutants of *Drosophila* Rabphilin exist to test its requirement, and other potential effectors for this particular function of Rab3 are unknown. Therefore, to explore the mechanism of Rab3 function further, we analyzed the traditional effector binding regions of Rab3 to determine residues that are required for Rab3 function at the *Drosophila* NMJ.

The main binding interface with which Rab3 interacts with its effectors includes two switch regions (Fig 1A, pink). Switch I is comprised of residues in the α1β2 loop and extends into the β2 strand; Switch II begins near the end of the β3 strand and extends through the α2 helix and into the α2β4 loop [23,29,44]. The switch regions undergo conformational changes that are dependent on the state of the guanine nucleotide that is bound and thus determine the proteins that Rab3 binds in each state [17,45]. Within the larger Rab family, the sequence and structure of the switch regions are important for determining the function of each Rab type as switch region variability between different Rabs is thought to contribute to effector protein binding specificity [46,47]. However, studies of mammalian Rab3 indicate that multiple effector proteins bind to overlapping residues in the switch regions [31–33,48], complicating attempts to define specific molecular interactions required for Rab3 function at the *Drosophila* NMJ by studying mutations in the switch regions. Therefore, rather than performing an exhaustive mutational analysis of the switch regions, we wished to determine more generally whether the switch regions are required for Rab3 to control active zone development while also identifying amino acids that may contribute to this function.

To test whether the switch regions are required for Rab3 function at the NMJ, we made single amino acid substitutions in the switch 1 and switch 2 regions, primarily analyzing amino acids within the α1β2 and β3α2 loops rather than residues in secondary structures to reduce the chances of altering Rab3 structure. To study the requirement of the switch 1 region, we generated the mutant transgenes *UAS-rab3F50A* and *UAS-rab3T53A* that code for mutations F50A and T53A respectively in the α1β2 loop and *UAS-rab3F58S* which codes for the mutation F58S in the β2 strand. Similarly, to study the necessity of residues within the switch 2 region, we generated the transgenes *UAS-rab3R82A* and *UAS-rab3Y83A* which code for mutations R82A and Y83A respectively in the β3α2 loop (note that the Q80L mutation is also within this loop). These mutations were designed with reference to prior analyses of mammalian Rab3 function. Studies of rodent Rab3 suggest that mutations equivalent to F50A andT53A reduce the affinity of Rab3 for Rab3-GEF [31]. Furthermore, Rabphilin binding is reduced by the mutation T53A and inhibited by the mutation F58S [32]. Less work has been done to determine the requirement of specific amino acids within the switch 2 region for effector binding. Crystal structure analysis indicates that the mammalian equivalents of R82 and Y83 are both contact sites for Rabphillin [34], although mutation of Y83 does not disrupt Rabphilin binding [33]. Y83 is also one of several residues that may interact with RIM [33], but its individual requirement is unclear.

**Fig 6:**
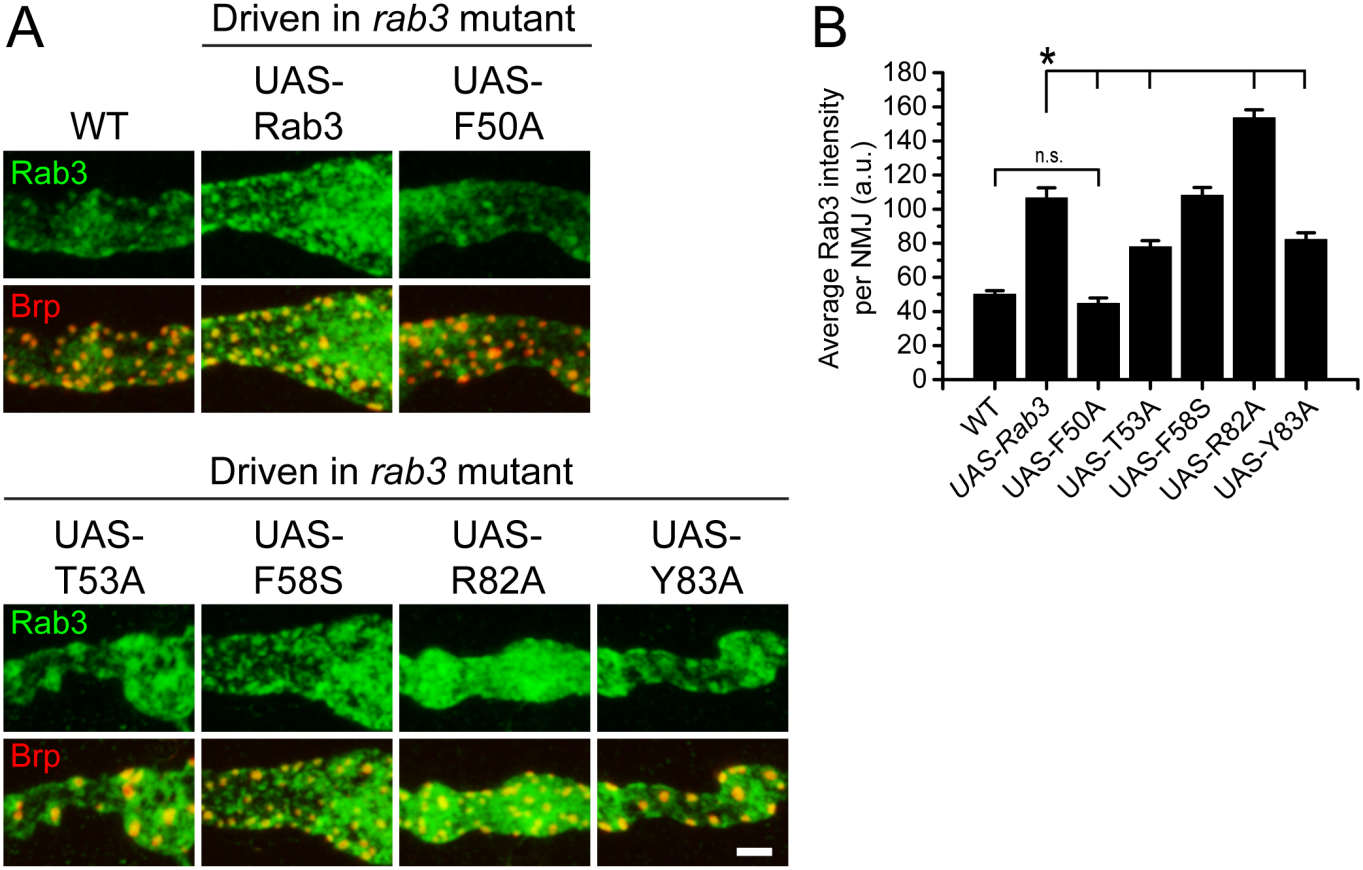
Switch region mutants traffick to NMJs with variable efficiency. (A) Images of NMJs costained with α-Rab3 (green) and α-Brp (red) from WT (*dvglut* ^NMJX^-*Gal4*/+), the *rab3* mutant expressing the wild type *UAS-rab3* transgene (*dvglut* ^NMJX^-*Gal4*/+; *rab3* ^*rup*^/Df(2R)ED2076; *UAS-rab3*/+), the *rab3* mutant expressing the *UAS-rab3F50A* transgene (*dvglut* ^NMJX^-*Gal4*/+; *rab3* ^*rup*^/Df(2R)ED2076; *UAS-rab3F50A*/+), the *rab3* mutant expressing the *UAS-rab3T53A* transgene (*dvglut* ^NMJX^-*Gal4*/+; *rab3* ^*rup*^/Df(2R)ED2076; *UAS-rab3T53A*/+), the *rab3* mutant expressing the *UAS-rab3F58S* transgene (*dvglut* ^NMJX^-*Gal4*/+; *rab3* ^*rup*^/Df(2R)ED2076; *UAS-rab3F58S*/+), the *rab3* mutant expressing the *UAS-rab3R82A* transgene (*dvglut* ^NMJX^-*Gal4*/+; *rab3* ^*rup*^/Df(2R)ED2076; *UAS-rab3R82A*/+), and the *rab3* mutant expressing the *UAS-rab3Y83A* transgene (*dvglut* ^NMJX^-*Gal4*/+; *rab3* ^*rup*^/Df(2R)ED2076; *UAS-rab3Y83A*/+). Scale bar, 2μm. (B) Histogram shows average intensity of Rab3 per NMJ for the genotypes listed in (A). n = 8 NMJs for all genotypes; *p<0.005.

To test the effect of switch region mutation on Rab3 function, we first examined whether the mutant Rab3 proteins are able to localize to the NMJ following expression via the *dvglut* ^NMJX^-*Gal4* driver in the *rab3* mutant background. α-Rab3 staining reveals that all five mutant proteins accumulate in the NMJ at levels equal to or greater than endogenous Rab3 (Fig 6). The F58S mutation has no effect on Rab3 trafficking, accumulation, and localization in NMJs, but the other mutations affect Rab3 localization to varying degrees. The F50A mutation causes the greatest defect in trafficking with the average intensity of Rab3F50A signal significantly decreased as compared to Rab3 expressed by the wild type *UAS-rab3* transgene. However, Rab3F50A signal is not significantly different from endogenous Rab3 and is still observed to aggregate at some active zones. Conversely, while the average intensities of Rab3T53A and Rab3Y83A at the NMJ are moderately reduced as compared to Rab3 driven by the *UAS-rab3* transgene, their levels are much greater than endogenous Rab3. Rab3T53A and Rab3Y83 also maintain the ability to cluster at Brp-positive sites. Interestingly, the R82A mutation actually enhances Rab3 accumulation and results in an increase in average Rab3 intensity per NMJ as compared to transgenically expressed wild type Rab3. Whereas the Rab3 staining pattern is usually mottled in appearance with greater accumulation in some areas of the NMJ than others, Rab3R82A signal is much more ubiquitous throughout the NMJ, seeming to fill the entire cytoplasmic space (Fig 6A).

**Fig 7:**
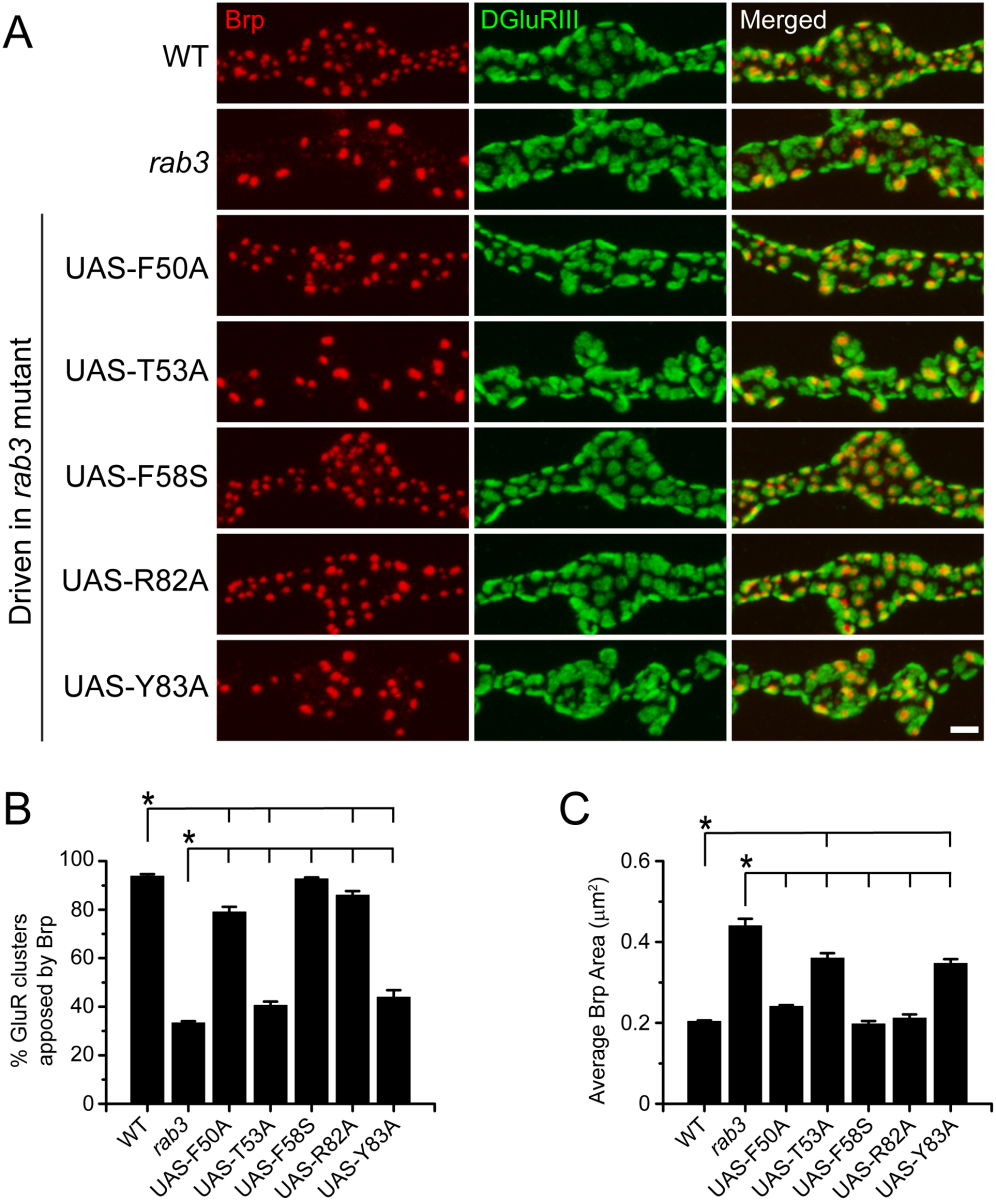
Switch region point mutations cause variable deficits in Rab3 function to control Brp distribution. (A) Images of NMJs costained with α-Brp (red) and α-DGluRIII (green) from WT (*dvglut* ^NMJX^-*Gal4*/+), the *rab3* mutant (*dvglut* ^NMJX^-*Gal4*/+; *rab3* ^*rup*^/Df(2R)ED2076), the *rab3* mutant expressing the *UAS-rab3F50A* transgene (*dvglut* ^NMJX^-*Gal4*/+; *rab3* ^*rup*^/Df(2R)ED2076; *UAS-rab3F50A*/+), the *rab3* mutant expressing the *UAS-rab3T53A* transgene (*dvglut* ^NMJX^-*Gal4*/+; *rab3* ^*rup*^/Df(2R)ED2076; *UAS-rab3T53A*/+), the *rab3* mutant expressing the *UAS-rab3F58S* transgene (*dvglut* ^NMJX^-*Gal4*/+; *rab3* ^*rup*^/Df(2R)ED2076; *UAS-rab3F58S*/+), the *rab3* mutant expressing the *UAS-rab3R82A* transgene (*dvglut* ^NMJX^-*Gal4*/+; *rab3* ^*rup*^/Df(2R)ED2076; *UAS-rab3R82A*/+), and the *rab3* mutant expressing the *UAS-rab3Y83A* transgene (*dvglut* ^NMJX^-*Gal4*/+; *rab3* ^*rup*^/Df(2R)ED2076; *UAS-rab3Y83A*/+). Scale bar, 2μm. (B-C) Histograms show (B) the average percentage of DGluRIII clusters apposed to Brp puncta per NMJ and (C) the average area of individual Brp puncta for the genotypes listed in (A). n = 8 NMJs for all genotypes; *p<0.001.

Since all switch region mutants accumulate in the NMJ at levels similar to or greater than endogenous Rab3, we next tested whether they could rescue the *rab3* mutant active zone phenotype by assaying Brp distribution. When expressed in the *rab3* mutant, all three switch 1 region mutants rescued the morphological synaptic phenotype, but the degree of rescue varied substantially among the mutant transgenes (Fig 7). Expression of Rab3F58S fully rescues the *rab3* mutant phenotype, resulting in NMJs identical to wild type in terms of both the percentage of GluR clusters apposed to Brp and average Brp puncta area. However, functional deficits are observed for Rab3F50A and Rab3T53A that incorporate mutations in the α1β2 loop of switch 1. Expression of Rab3F50A in the *rab3* mutant produces a strong but incomplete rescue wherein approximately 20% of GluR clusters remain unapposed to Brp but average Brp size is similar to wild type. Conversely, expression of Rab3T53A results in a weak rescue with about 60% of GluR clusters remaining unapposed to Brp and Brp size only moderately reduced as compared to the *rab3* mutant. To ensure that defective Rab3 function is due to the specific mutation of the F50 and T53 residues rather than general alterations in the α1β2 loop we also analyzed the effect of mutating the neighboring α1β2 loop residues V51 and S52. We find that V51A and S52A mutations do not disrupt Rab3 function as expression of Rab3V51A and Rab352A in the *rab3* mutant results in NMJs identical to wild type in terms of the percentage of GluR clusters apposed to Brp (WT, 93.8 ± 0.9%; Rab3V51A expressed in *rab3* ^*rup*^/Df(2R)ED2076, 93.6 ± 0.7%; Rab3S52A expressed in *rab3* ^*rup*^/Df(2R)ED2076, 93.7 ± 1.1%, n = 8 NMJs for all genotypes; p>0.9 for WT vs. both Rab3V51A and Rab3S52A) and average Brp puncta area (WT, 0.20 ± 0.003 μm^2^; Rab3V51A expressed in *rab3* ^*rup*^/Df(2R)ED2076, 0.20 ± 0.004 μm^2^; Rab3S52A expressed in *rab3* ^*rup*^/Df(2R)ED2076, 0.20 ± 0.007 μm^2^, n = 8 NMJs for all genotypes; p>0.8 for WT vs. both Rab3V51A and Rab3S52A). These findings indicate a specific requirement for F50 and T53 for proper switch 1 region interactions at the NMJ.

The effects of mutations within the β3α2 loop of switch 2 are similarly variable. Expression of Rab3R82A and Rab3Y83A in the *rab3* mutant each produces a partial rescue of Brp distribution (Fig 7). However, whereas Rab3R82A results in full rescue of Brp puncta size and a strong but incomplete rescue of Brp/GluR apposition, Rab3Y83A expression results in a weak rescue of both Brp puncta size and apposition that is comparable to the degree of rescue observed following Rab3T53A expression. Taken together, these results indicate that both switch regions are required for proper Rab3 function at the NMJ and that the T53 and Y83 residues are of particular significance. Furthermore, since the F58S mutation inhibits Rabphilin binding to rodent Rab3, the fact that Rab3F58S can fully rescue the *rab3* mutant phenotype suggests that Rab3philin is not required for Rab3 function to control active zone composition.

We previously showed that Rab3T35N and Rab3N134I not only lacked the ability to rescue the *rab3* mutant phenotype but also acted as dominant negatives when expressed in a wild type background (Fig 3), potentially by sequestering Rab3-GEF as has been postulated by homologous variants of mammalian Rab3 [31]. Since the T53A and Y83A mutations disrupt Rab3 function, we also tested whether Rab3T53A and Rab3Y83A have dominant negative properties. NMJs from wild type larvae that express Rab3T53A and Rab3Y83A driven by ELAV-*GeneSwitch* appear similar to wild type with normal Brp apposition and size (S1 Fig). Thus, the T53A and Y83A mutations are more likely to disrupt protein interactions with Rab3 than result in the sequestration of essential factors.

### Mutations within the CDR Regions do not Disrupt Rab3 Function

Analysis of the crystal structure of Rab3 bound to Rabphilin indicates that binding involves the interaction of Rabphilin with both the switch regions and a separate binding pocket that is lined by three complementarity-determining regions (CDRs) [34]. The sequences of these three RabCDRs (Fig 1A, yellow) vary among Rab family members, leading to the hypothesis that the CDRs mediate interaction specificity between a Rab and its effectors [34,47]. Previous studies also indicate that the CDR regions may be a necessary component of effector interaction as deletion of the first CDR eliminates the ability of Rab3 to bind Rabphilin [34]. Therefore, to further examine which elements of the Rab3 effector binding interface are required for Rab3 function at the NMJ, we next tested whether mutations made within the CDR regions disrupt Rab3 function.

We generated *UAS-rab3* transgenes that code for alanine substitutions within each of the three CDRs. Since little is known about the interaction between the Rab3CDRs and other effectors, we designed the CDR mutations to specifically disrupt residues observed to interact with Rabphilin [34], assuming that similar residues may be involved in the binding of multiple effectors. This allowed us to make more discrete changes to Rab3 and avoid larger mutations covering entire CDR sequences that may result in protein misfolding. To disrupt the first CDR located within the unstructured region just N-terminal to the first β-sheet, we constructed the *UAS-rab3FDY18-20AAA* transgene that codes for mutation of the three residues required for Raphilin binding. Likewise, to disrupt the second CDR, we generated the *UAS-rab3WDN124-126AAA* transgene that results in the mutation of the majority of the α3β5 loop. The third CDR is primarily located within the final α-helix. To study the requirement of the third CDR while also avoiding changes in protein folding, we created two mutant transgenes, each coding for the mutation of two residues reported to interact with Rabphilin: *UAS-rab3KM185-186AA* and *UAS-rab3SL189-190AA*.

**Fig 8:**
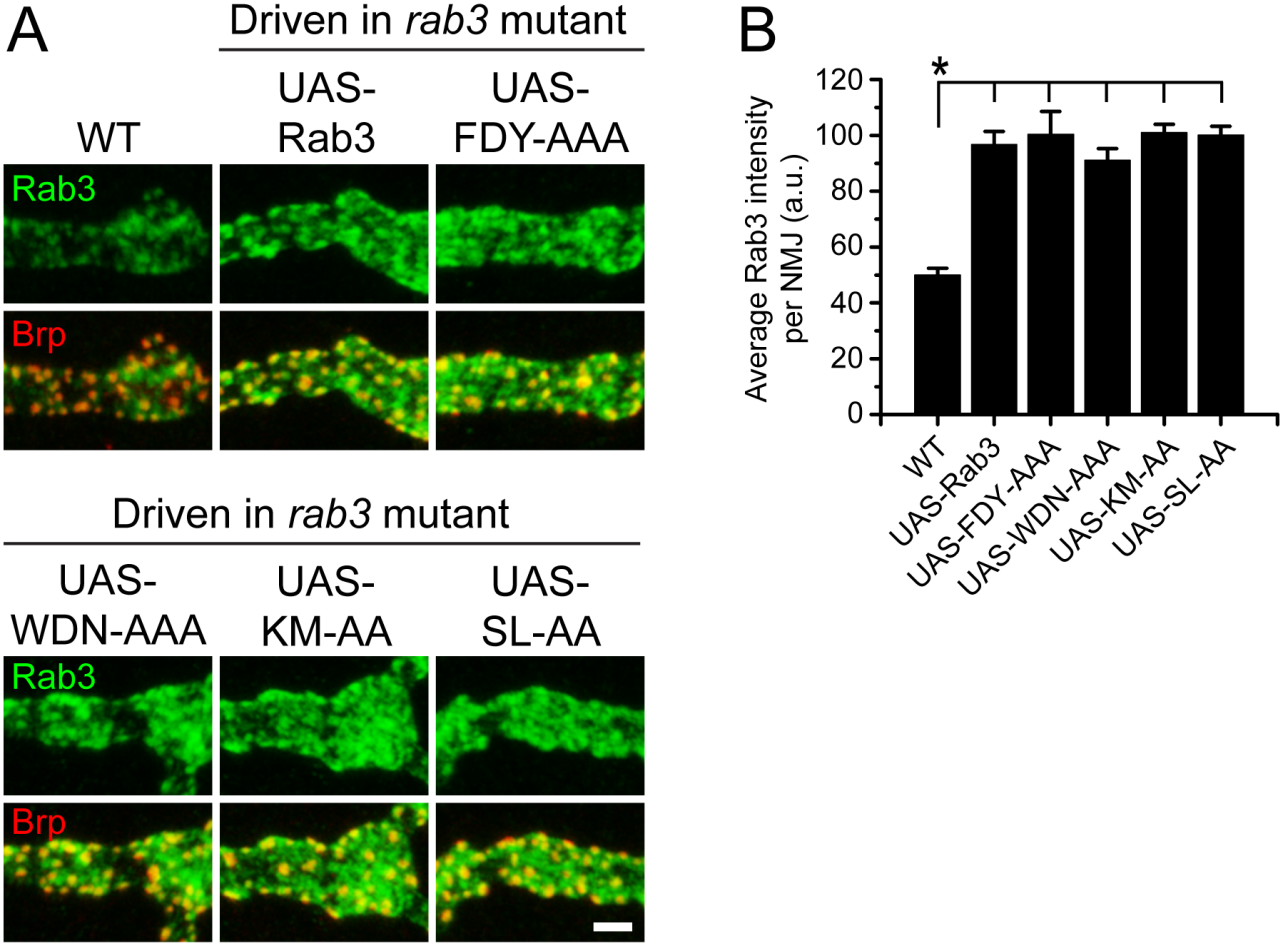
CDR region mutations do not disrupt Rab3 trafficking to NMJs. (A) Images of NMJs costained with α-Rab3 (green) and α-Brp (red) from WT (*dvglut* ^NMJX^-*Gal4*/+), the *rab3* mutant expressing the wild type *UAS-rab3* transgene (*dvglut* ^NMJX^-*Gal4*/+; *rab3* ^*rup*^/Df(2R)ED2076; *UAS-rab3*/+), the *rab3* mutant expressing the *UAS-rab3FDY18-20AAA* transgene (*dvglut* ^NMJX^-*Gal4*/+; *rab3* ^*rup*^/Df(2R)ED2076; *UAS-rab3FDY18-20AAA*/+), the *rab3* mutant expressing the *UAS-rab3WDN124-126AAA* transgene (*dvglut* ^NMJX^-*Gal4*/+; *rab3* ^*rup*^/Df(2R)ED2076; *UAS-rab3WDN124-126AAA*/+), the *rab3* mutant expressing the *UAS-rab3KM185-186AA* transgene (*dvglut* ^NMJX^-*Gal4*/+; *rab3* ^*rup*^/Df(2R)ED2076; *UAS-rab3KM185-186AA*/+), and the *rab3* mutant expressing the *UAS-rab3SL189-190AA* transgene (*dvglut* ^NMJX^-*Gal4*/+; *rab3* ^*rup*^/Df(2R)ED2076; *UAS-rab3SL189-190AA*/+). Scale bar, 2μm. (B) Histogram shows average intensity of Rab3 per NMJ for the genotypes listed in (A). n = 8 NMJs for all genotypes; *p<<0.001.

**Fig 9:**
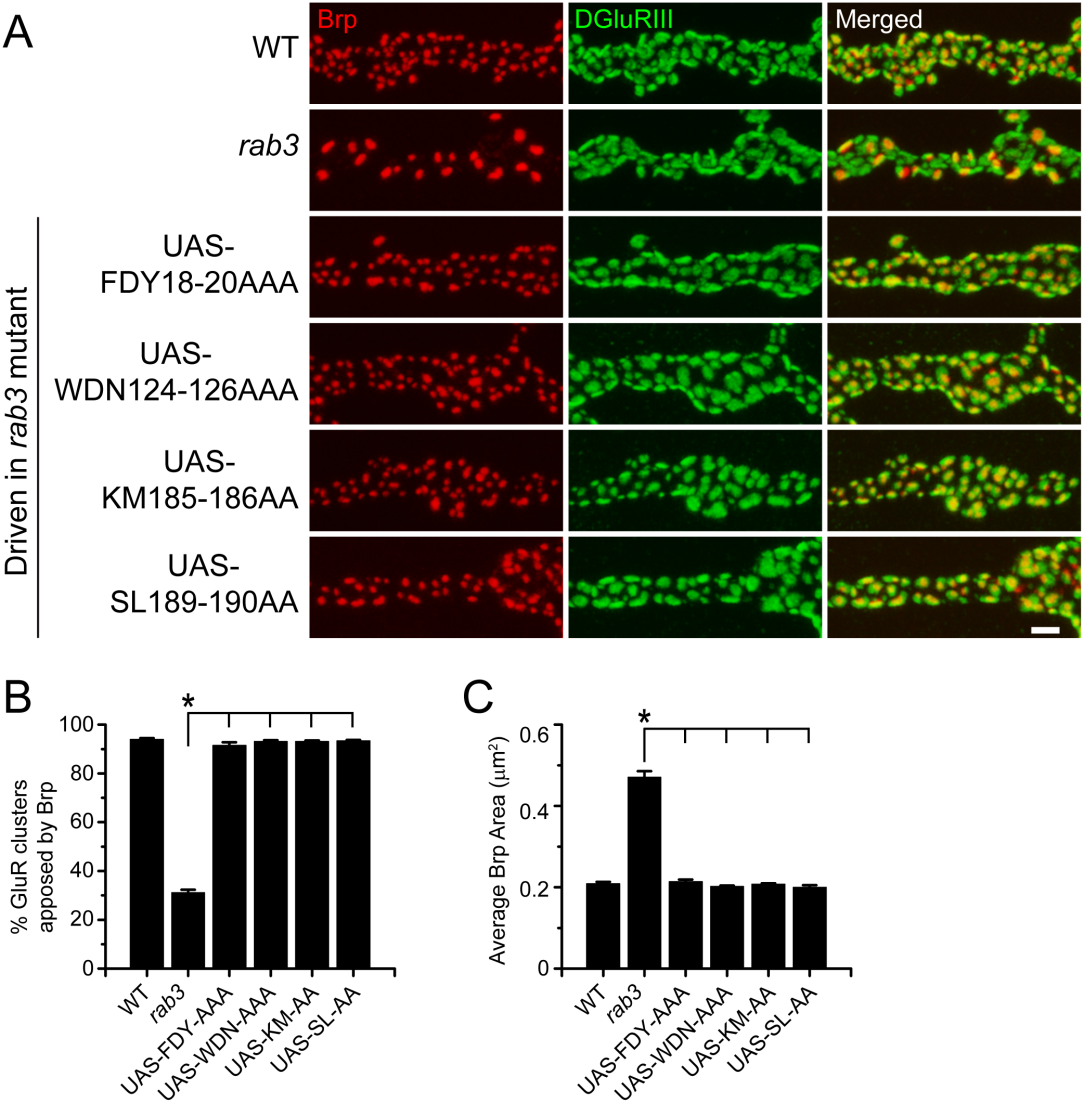
CDR region mutations do not impair Rab3 function to control Brp distribution. (A) Images of NMJs costained with α-Brp (red) and α-DGluRIII (green) from WT (*dvglut* ^NMJX^-*Gal4*/+), the *rab3* mutant (*dvglut* ^NMJX^-*Gal4*/+; *rab3* ^*rup*^/Df(2R)ED2076), the *rab3* mutant expressing the *UAS-rab3FDY18-20AAA* transgene (*dvglut* ^NMJX^-*Gal4*/+; *rab3* ^*rup*^/Df(2R)ED2076; *UAS-rab3FDY18-20AAA*/+), the *rab3* mutant expressing the *UAS-rab3WDN124-126AAA* transgene (*dvglut* ^NMJX^-*Gal4*/+; *rab3* ^*rup*^/Df(2R)ED2076; *UAS-rab3WDN124-126AAA*/+), the *rab3* mutant expressing the *UAS-rab3KM185-186AA* transgene (*dvglut* ^NMJX^-*Gal4*/+; *rab3* ^*rup*^/Df(2R)ED2076; *UAS-rab3KM185-186AA*/+), and the *rab3* mutant expressing the *UAS-rab3SL189-190AA* transgene (*dvglut* ^NMJX^-*Gal4*/+; *rab3* ^*rup*^/Df(2R)ED2076; *UAS-rab3SL189-190AA*/+). Scale bar, 2μm. (B-C) Histograms show (B) the average percentage of DGluRIII clusters apposed to Brp puncta per NMJ and (C) the average area of individual Brp puncta for the genotypes listed in (A). n = 8 NMJs for all genotypes; *p<<0.001.

Prior to testing whether mutation of the CDR regions disrupts Rab3 function, we analyzed whether the CDR mutants localize properly to the NMJ. Expression of the mutated transgenes with the *dvglut* ^NMJX^-*Gal4* driver in the *rab3* mutant reveals normal axonal trafficking for all four CDR mutants (Fig 8). Rab3FDY18-20AAA, Rab3WDN124-126, Rab3KM185-186AA, and Rab3SL189-190AA all exhibit normal α-Rab3 staining patterns at the NMJ and aggregate at Brp-positive sites (Fig 8A). In addition, average intensity of Rab3 signal at the NMJ is similar for neurons expressing the CDR mutant transgenes and neurons expressing the wild type transgene and greater than endogenous Rab3 (Fig 8B). Since Rab3 localization is not affected by mutation of the CDR regions, we next examined the ability of the CDR mutants to rescue the Brp distribution phenotype of the *rab3* mutant. Rab3FDY18-20AAA, Rab3WDN124-126, Rab3KM185-186AA, and Rab3SL189-190AA all fully rescue the *rab3* mutant phenotype (Fig 9), resulting in measurements of GluR-Brp apposition and Brp size that are identical to wild type (Fig 9B and 9C). Thus, while we did not examine every residue, we were unable to identify any specific amino acids within the CDR regions that are required for Rab3 function in the control of active zone composition.

### Residues Required for Membrane Attachment are Necessary for Rab3 Function

As discussed previously, the primary function of Rab proteins is vesicle trafficking and tethering at target membranes [17]. In the synaptic vesicle cycle, Rab3 docks neurotransmitter-filled synaptic vesicles at release sites through direct attachment to vesicle membranes [19]. Experiments that alter synaptic vesicle release have no effect on Brp distribution at WT or *rab3* mutant NMJs [14]. However, the possibility remains that Rab3 controls active zone composition via a vesicle docking mechanism. To determine whether vesicle attachment is required for Rab3 to control active zone development, we asked whether preventing membrane association disrupts Rab3 function. Vesicle association requires the posttranslational addition of a geranylgeranyl moiety to the CXC motif at the carboxyl-terminus of Rab3 [49]. C-terminal truncation of the protein that removes the last three amino acids prevents both lipid modification and membrane attachment [28]. Thus, to test the requirement of vesicle binding, we generated *UAS-rab3ΔC*, which codes for a truncated Rab3 protein that lacks the last three amino acids, and examined whether its expression can rescue the *rab3* mutant synaptic phenotype.

**Fig 10:**
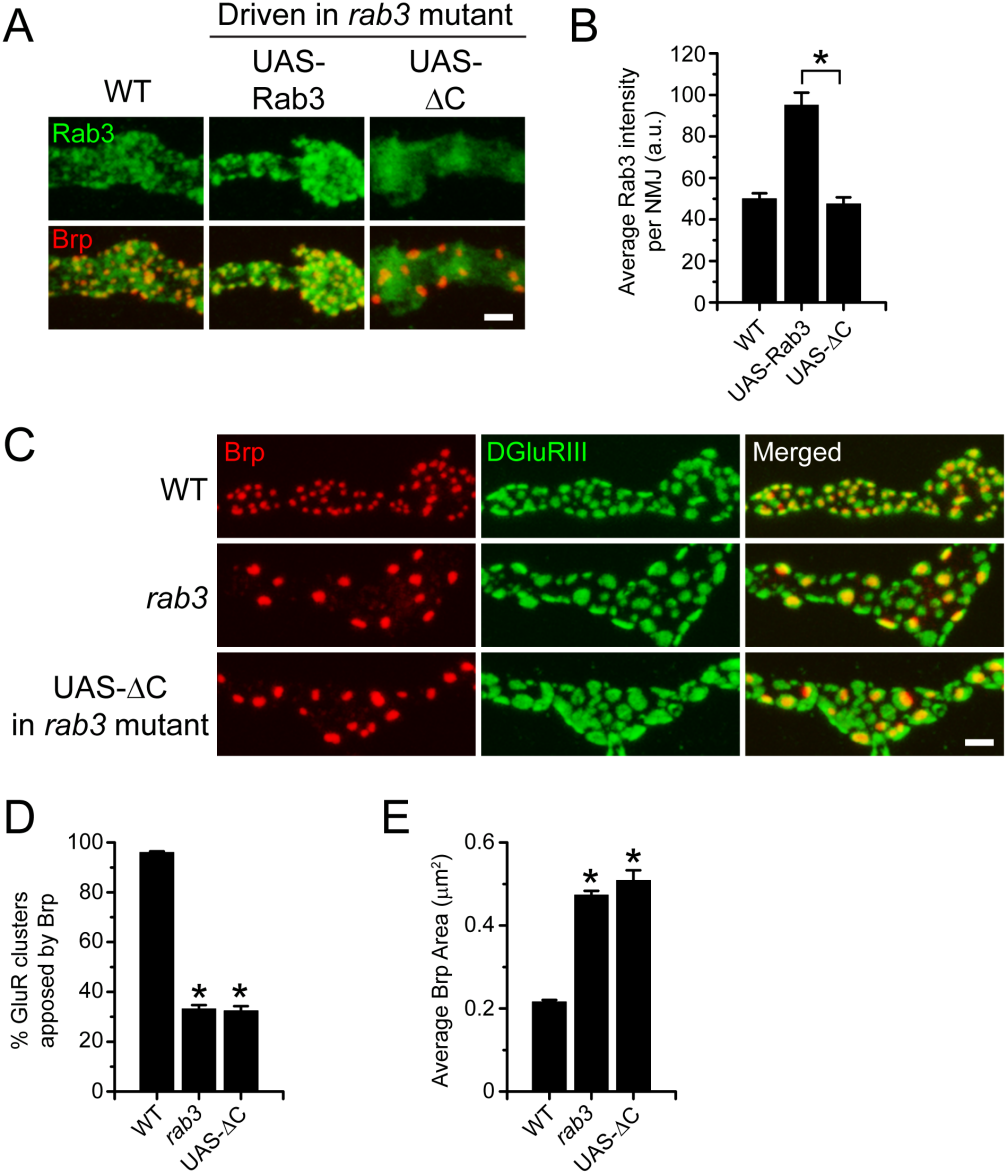
Truncation that prevents membrane attachment impairs Rab3 trafficking and renders it nonfunctional to control Brp distribution. (A) Images of NMJs costained with α-Brp (red) and α-DGluRIII (green) from WT (*dvglut* ^NMJX^-*Gal4*/+), the *rab3* mutant expressing the wild type *UAS-rab3* transgene (*dvglut* ^NMJX^-*Gal4*/+; *rab3* ^*rup*^/Df(2R)ED2076; *UAS-rab3*/+), and the *rab3* mutant expressing the *UAS-rab3 UAS-rab3ΔC* transgene (*dvglut* ^NMJX^-*Gal4*/+; *rab3* ^*rup*^/Df(2R)ED2076; *UAS-rab3ΔC*/+). Scale bar, 2μm. (B) Histogram shows average intensity of Rab3 per NMJ for the genotypes listed in (A). n = 10 NMJs for all genotypes; *p<<0.001. (C) Images of NMJs costained with α-Brp (red) and α-DGluRIII (green) from WT (*dvglut* ^NMJX^-*Gal4*/+), the *rab3* mutant (*dvglut* ^NMJX^-*Gal4*/+; *rab3* ^*rup*^/Df(2R)ED2076), and the *rab3* mutant expressing the *UAS-rab3ΔC* transgene (*dvglut* ^NMJX^-*Gal4*/+; *rab3* ^*rup*^/Df(2R)ED2076; *UAS-rab3ΔC*/+). Scale bar, 2μm. (D-E) Histograms show (D) the average percentage of DGluRIII clusters apposed to Brp puncta per NMJ and (E) the average area of individual Brp puncta for the genotypes listed in (C). n = 10 NMJs for all genotypes; *p<<0.001 versus WT.

α-Rab3 staining reveals that when expressed via the *dvglut* ^NMJX^-*Gal4* driver in the *rab3* mutant, Rab3ΔC accumulates at the NMJ, although C-terminal truncation does result in localization and trafficking defects (Fig 10A and 10B). Rab3ΔC fails to aggregate in discrete puncta that colocalize with Brp but instead localizes in a more diffuse pattern throughout the NMJ (Fig 10A). Furthermore, the average intensity of Rab3ΔC at the NMJ is significantly lower than wild type Rab3 expressed by the *UAS-rab3* transgene (Fig 10B). However, the average intensity of Rab3ΔC staining is similar to endogenous Rab3 (p>0.9), despite the observed localization defects (Fig 10B). Since Rab3ΔC is present in the NMJ at levels comparable to endogenous Rab3, we next tested whether it can rescue Brp distribution in the *rab3* mutant. Co-staining with α-Brp and α-DGluRIII reveals a complete lack of rescue when *UAS-rab3ΔC* is expressed in the mutant (Fig 10C). No difference is observed between *rab3* mutant NMJs and mutant NMJs expressing *UAS-rab3ΔC*, both in terms of the percentage of GluR clusters apposed to Brp (Fig 10D) and average Brp size (Fig 10E). Furthermore, Rab3ΔC is unlikely to sequester essential factors required for Rab3 function as expression of *UAS-rab3ΔC* in a wild type background via ELAV-*GeneSwitch* fails to produce a dominant negative effect (S1 Fig). Since disruption of membrane attachment results in defective Rab3 function at the NMJ, these results suggest that Rab3 controls the distribution of active zone components via a vesicle associated mechanism.

## Discussion

Rab3 has been implicated in a novel function that controls the protein composition of active zones [14]. To investigate the mechanism of Rab3 function at the *Drosophila* NMJ, we conducted a mutational analysis of Rab3 to determine its molecular and structural requirements for the control of active zone development. We show that GTP-binding is essential for proper trafficking of Rab3 to NMJs and for Rab3 to distribute Brp across release sites. Conversely, the hydrolysis of bound GTP to GDP is not required for Rab3 to control NMJ morphology or function, although expression of constitutively active Rab3 results in a mild reduction in Brp cluster size. Structure-function analysis of the effector-binding interface indicates a requirement for specific residues in the switch regions previously identified for their involvement in protein-protein interactions. Furthermore, deletion of the residues necessary for membrane insertion disrupts Rab3 function, revealing a requirement for vesicle attachment. Our findings are comparable to previous studies of Rab3 function and suggest that Rab3 controls active zone composition by a vesicle associated mechanism that is typical for Rab proteins.

### GTP/GDP Cycling

Rab3 alternates between a GTP-bound state and a GDP-bound state in a cycle that determines its interaction with effector proteins [17]. Consistent with previous studies of mammalian Rab3 function, GTP-binding is required for Rab3 function in *Drosophila* neurons. Both Rab3N134I and Rab3T35N, variants that are defective in their GTP-binding properties, are unable to rescue the *rab3* mutant phenotype, likely due to several related deficits. Rab3N134I and Rab3T35N fail to localize properly to NMJs. In rodent neurons, anterograde transport of Rab3 to axon terminals requires direct interaction with Rab3-GEF, which acts as a structural linker between Rab3 and kinesin motor proteins [36]. Transport via this mechanism occurs more efficiently when Rab3 is in its GTP-bound state. While it is unclear if a similar transport mechanism is utilized in *Drosophila* neurons, our findings are consistent with previous work. Importantly, Rab3T35N protein accumulates in NMJs regardless of defective transport but still fails to rescue Brp distribution in the *rab3* mutant, indicating that GTP-binding is required for Rab3 function at the NMJ itself. However, Rab3N134I and Rab3T35N also act as dominant negatives, producing a hypomorphic *rab3* phenotype when driven in wild type neurons. Studies of mammalian Rab3 indicate that Rab3N134I and Rab3T35N inhibit endogenous Rab3 function by sequestering Rab3-GEF [31,37]. Thus, the inability of Rab3N134I and Rab3T35N to rescue Brp distribution in *rab3* mutant NMJs is likely caused by multiple mechanistic defects. Interestingly, overexpression of Rab3N134I and Rab3T35N in wild type neurons disrupts Brp distribution only in NMJs associated with posterior segments. Since enhanced phenotypic severity in posterior NMJs is a trait generally observed in axon transport mutants [50,51], further studies analyzing the axonal trafficking of Brp and other synaptic proteins will be useful for determining whether the overexpression of variant forms of Rab3 cause general axonal transport defects.

Whereas GTP-binding is required for Rab3 function at the NMJ, hydrolysis-defective Rab3Q80L can rescue active zone composition in *rab3* mutants. Expression of Rab3Q80L in *rab3* mutant neurons results in the distribution of Brp to all release sites and decreased Brp cluster size. Rab3Q80L expression also rescues the short-term facilitation defects of the *rab3* mutant, likely by distributing CAZ components across all active zones. Thus, while GTP-binding is essential, the cycling of guanine nucleotides and the inactivation of Rab3 to its GDP-bound form are not required for Rab3 to control active zone development. These results are similar to mammalian studies that show little functional difference between wild type Rab3 and the Q80L variant for exocytosis [38,52,53]. Interestingly, at *Drosophila* NMJs expression of Rab3Q80L also results in a mild gain-of-function effect, causing a moderate but significant reduction in average Brp cluster size as compared to wild type. This gain-of-function effect is observed regardless of whether Rab3Q80L is expressed in wild type or *rab3* mutant neurons. The reason for this effect is unclear; however, it may be due to the constitutively active nature of Rab3Q80L. Since Rab3Q80L is primarily locked in a GTP-bound state [30], its dissociation from effectors may be attenuated and result in their sequestration. A similar phenotype is not observed in *rab3-GAP* mutants, although Rab3 is unlikely to be GTP-locked in *rab3-GAP* mutant NMJs as the GAP enhances the hydrolytic activity of Rab3 but is not a requirement for GTP-hydrolysis [35]. Despite the modest morphological gain-of-function effect, evoked EJCs, spontaneous mEJCs, and short-term facilitation are similar between wild type larvae and *rab3* mutant larvae expressing Rab3Q80L. Thus, Rab3Q80L expression results in normal NMJ function.

### Effector Binding Regions

Rab3 binds other proteins via an interface composed of two switch regions and a CDR binding pocket. Biochemical and structural studies of mammalian Rab3 indicate that the switch regions play a prominent role in interactions with regulatory and effector proteins and that the CDR regions may determine effector specificity [34,45]. Consistent with these studies, our structure-function analysis reveals that specific amino acids within the switch regions are required for Rab3 to control Brp distribution across active zones. Conversely, we were unable to identify any amino acids within the CDR regions required for Rab3 function or accumulation at NMJs.

How do protein-protein interactions facilitated by the switch regions relate to Rab3 function in the control of active zone composition? The mutations F50A and T53A in switch 1 both disrupt Rab3 localization and function to varying degrees. Whereas the F50A mutation causes a substantial reduction in Rab3 accumulation at the NMJ, Rab3F50A expression in *rab3* mutant neurons results in a strong albeit incomplete rescue of Brp distribution. In contrast, Rab3T53A accumulation is only modestly reduced as compared to transgenic wild type Rab3, but its expression in the mutant results in minimal rescue. Studies of comparable mutations in mammalian Rab3 indicate that both F50A and T53A decrease the binding affinity of Rab3 to Rab3-GEF [31]. It is unsurprising that mutations which disrupt Rab3-GEF interaction may lead to defective Rab3 trafficking and function at the NMJ. Since GEF activity catalyzes GDP to GTP exchange, defective Rab3-GEF binding likely results in a reduced GTP-bound population of Rab3. Moreover, decreased affinity for Rab3-GEF may disrupt the Rab3/Rab3-GEF/kinesin linkage required for efficient transport [36]. However, neither the F50A or T53A mutations result in trafficking and functional deficits as severe as we observe for the GTP-binding defective variants Rab3N134I and Rab3T35N, which may be comparable to a loss of GEF function. Furthermore, the differential localization and functional deficits of Rab3F50A and Rab3T53A suggest that the observed results may not be solely due to defective Rab3-GEF binding. Studies of mammalian Rab3 and Ras indicate a role for T53 in the binding and coordination of the Mg^2+^ associated with the guanine nucleotide [48,54], and mutations equivalent to T53A also disrupt Rabphilin binding [32]. Thus, this residue is likely involved in the proper binding of multiple factors. Nevertheless, the fact that Rab3T53A trafficks well to NMJs and aggregates in Brp-positive regions in a manner similar to wild type Rab3 suggests that the T53A mutation does not result in global loss of Rab3 function as is observed for N134I and T35N variants. Rather, T53A mutation may cause a more discrete defect such as ineffective binding of an effector protein in the NMJ itself.

The functional deficits associated with the Y83A mutation in switch 2 are remarkably similar to those observed for T53A. Rab3Y83A aggregates in Brp-positive regions and trafficks well with only a minor reduction in protein accumulation at the NMJ. However, expression of Rab3Y83A in *rab3* mutant neurons results in negligible rescue. Protein interactions that require the Y83 residue have not been defined, although the equivalent residue in mammalian Rab3 has been implicated in interactions with RIM and Rabphilin [33,34], suggesting that it may be required for effector binding at the NMJ. Interestingly, mutation of the neighboring R82 residue results in increased accumulation of Rab3 in NMJs with minimal effect on Rab3 function. It is unlikely that the augmented levels of Rab3R82A protein are due to enhanced transcription as expression variability was controlled for by direct insertion of all transgenes into the same genomic locus. Rather, the R82A mutation may disrupt protein interactions that control Rab3 localization.

The effector proteins that Rab3 must bind to control active zone composition remain unknown; however, our analysis indicates that Rabphilin is not required for Rab3 function. Mutations of mammalian Rab3 equivalent to F58S in the switch 1 region and FDY18-20AAA in the first CDR region prevent Rabphilin binding [32,34], but Rab3F58S and Rab3FDY18-20AAA traffick normally and fully rescue Brp distribution when expressed in the *rab3* mutant. Further work will be necessary for defining the Rab3 effectors required for active zone development. The identification of T53A and Y83A as mutations that primarily affect Rab3 function to control Brp distribution rather than Rab3 localization to the NMJ may be informative for future biochemical studies concerning identification of effector proteins involved in this mechanism.

### Membrane Association and Vesicle Docking

Conventional Rab protein function involves direct membrane attachment for the translocation and docking of vesicles [17]. Our findings are consistent with a model wherein Rab3 controls active zone development via a typical vesicle tethering mechanism. Truncation that prevents lipid modification and membrane association results in a loss of Rab3 function even though the protein accumulates in NMJs at levels similar to endogenous Rab3. Nevertheless, the type of vesicle with which Rab3 associates in this mechanism is unclear. The role of Rab3 in the regulation of neurotransmitter-filled synaptic vesicles at release sites is well established [19,21]. However, general perturbations in synaptic vesicle release do not affect Brp distribution at wild type or *rab3* mutant NMJs [14], reducing the likelihood that the docking of synaptic vesicles themselves is involved in this mechanism. On the other hand, recent studies reveal that Brp distribution is also altered following knockdown of Synaptotagmin-1 (Syt) [55], a key vesicle protein that controls Ca^2+^-triggered synaptic vesicle fusion [19]. *syt* knockdown reduces the number of Brp clusters, particularly in terminal boutons, and results in a moderate increase in Brp cluster size [55]. While the *syt* ^*KD*^ phenotype is not identical to that observed at *rab3* mutant NMJs, the identification of a second synaptic vesicle protein required for proper Brp distribution suggests that a potential linkage may exist between synaptic vesicle dynamics and CAZ assembly. Alternatively, Rab3 may be associated with a second vesicle population in *Drosophila* neurons and function via a mechanism that does not involve neurotransmitter-filled synaptic vesicles. Interestingly, in mammals, Rab3a/c is also a component of Piccolo-Bassoon Transport Vesicles (PTVs) [56], vesicles that carry a preassembled set of CAZ proteins for incorporation at developing synapses [57]. While PTVs have not been identified in *Drosophila*, a potential mechanism of Rab3 function to nucleate CAZ assembly may be the docking of analogous transport vesicles at nascent release sites. Understanding Rab3 function at the NMJ will require further work to determine the vesicles and effector proteins that Rab3 must bind to control active zone development.

## Supporting Information

S1 Fig Rab3T53A, Rab3Y83A, and Rab3ΔC do not produce a dominant negative phenoypte when expressed in wild type neurons. Images of NMJs costained with α-Brp (red) and α-DGluRIII (green) from WT (ELAV-*GeneSwitch*/+), the *UAS-rab3T53A* transgene expressed in a wild type background (ELAV-*GeneSwitch*/*UAS-rab3T53A*), the *UAS-rab3Y83A* transgene expressed in a wild type background (ELAV-*GeneSwitch*/*UAS-rab3Y83A*), and the *UAS-rabΔC* transgene expressed in a wild type background (ELAV-*GeneSwitch*/*UAS-rab3ΔC*), Scale bar, 2μm.
(TIF)
