# Supplementary figures and images for: Mutational Analysis of Rab3 Function for Controlling Active Zone Protein Composition at the Drosophila Neuromuscular Junction
Source: PLoS One. 2015 Aug 28;10(8):e0136938. doi: 10.1371/journal.pone.0136938 (PMC4552854; doi:10.1371/journal.pone.0136938)

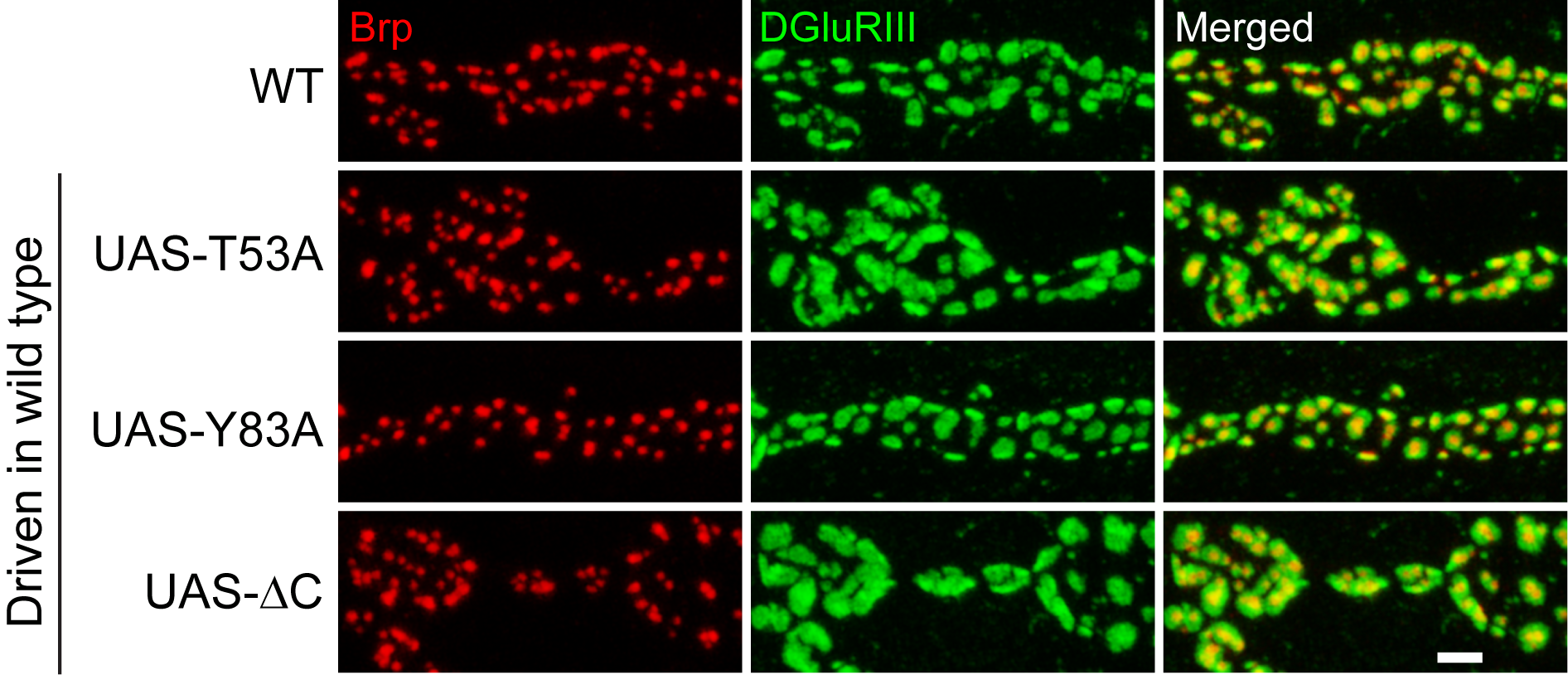

Supplement: S1 Fig — Images of NMJs costained with α-Brp (red) and α-DGluRIII (green) from WT (ELAV-GeneSwitch/+), the UAS-rab3T53A transgene expressed in a wild type background (ELAV-GeneSwitch/UAS-rab3T53A), the UAS-rab3Y83A transgene expressed in a wild type background (ELAV-GeneSwitch/UAS-rab3Y83A), and the UAS-rabΔC transgene expressed in a wild type background (ELAV-GeneSwitch/UAS-rab3ΔC), Scale bar, 2μm. (TIF) [file pone.0136938.s001.tif]
